# The European sea bass *Dicentrarchus labrax *genome puzzle: comparative BAC-mapping and low coverage shotgun sequencing

**DOI:** 10.1186/1471-2164-11-68

**Published:** 2010-01-27

**Authors:** Heiner Kuhl, Alfred Beck, Grzegorz Wozniak, Adelino VM Canario, Filip AM Volckaert, Richard Reinhardt

**Affiliations:** 1 Max Planck Institute for Molecular Genetics, Ihnestr. 63, D-14195 Berlin, Germany; 2 Centre of Marine Sciences (CCMAR), University of Algarve, Gambelas, 8005-139 Faro, Portugal; 3 Laboratory of Animal Diversity and Systematics, Katholieke Universiteit Leuven, Ch. Deberiotstraat 32, B-3000 Leuven, Belgium

## Abstract

**Background:**

Food supply from the ocean is constrained by the shortage of domesticated and selected fish. Development of genomic models of economically important fishes should assist with the removal of this bottleneck. European sea bass *Dicentrarchus labrax *L. (Moronidae, Perciformes, Teleostei) is one of the most important fishes in European marine aquaculture; growing genomic resources put it on its way to serve as an economic model.

**Results:**

End sequencing of a sea bass genomic BAC-library enabled the comparative mapping of the sea bass genome using the three-spined stickleback *Gasterosteus aculeatus *genome as a reference. BAC-end sequences (102,690) were aligned to the stickleback genome. The number of mappable BACs was improved using a two-fold coverage WGS dataset of sea bass resulting in a comparative BAC-map covering 87% of stickleback chromosomes with 588 BAC-contigs. The minimum size of 83 contigs covering 50% of the reference was 1.2 Mbp; the largest BAC-contig comprised 8.86 Mbp. More than 22,000 BAC-clones aligned with both ends to the reference genome. Intra-chromosomal rearrangements between sea bass and stickleback were identified. Size distributions of mapped BACs were used to calculate that the genome of sea bass may be only 1.3 fold larger than the 460 Mbp stickleback genome.

**Conclusions:**

The BAC map is used for sequencing single BACs or BAC-pools covering defined genomic entities by second generation sequencing technologies. Together with the WGS dataset it initiates a sea bass genome sequencing project. This will allow the quantification of polymorphisms through resequencing, which is important for selecting highly performing domesticated fish.

## Background

Teleost fishes are the most diverse group of vertebrates, with approximately 28,000 species, which have colonized a range of aquatic environments and display a variety of biochemical, physiological and morphological adaptations [1,2]. Because of this diversity and their position at the base of the vertebrate phylogeny, some species are considered good models of evolution, development and human diseases [3-6]. For this reason, teleost species were among the first vertebrate genomes to be sequenced: the green spotted pufferfish, *Tetraodon nigroviridis *[7]and the fugu *Takifugu rubripes *[8] for their relatively small compact genome; the medaka *Oryzias latipes *[9] and the zebrafish *Danio rerio *[10] for their value as developmental models, short life cycle, ease of maintenance and amenity to genetic manipulations [11,12]; and the three-spined stickleback, *Gasterosteus aculeatus *http://www.ensembl.org as a model for evolution [13]. However, no representative of the Perciformes, the most advanced and diverse group of teleosts has been sequenced and genomic resources for this taxonomic group are relatively limited. Furthermore, no aquaculture fish species has had its genome sequenced until now. Although sequences from model teleost fish genomes are a valuable tool for comparative approaches to elucidate the genomics of phylogenetically related non-model teleost [14-17], they are selected for the opposite reasons of aquaculture species, which generally have large body mass and long reproductive cycles.

The European sea bass *Dicentrarchus labrax *L. (Moronidae, Perciformes, Teleostei) is a major fisheries and aquaculture species in the Mediterranean and Atlantic coasts of Europe and North Africa. Its industrial production has steadily grown over the past two decades and in 2008 it reached at least 105,900 metric tonnes http://www.globefish.org. Worldwide, basses and other perciform fish, which include the tunas, breams and tilapias, account for over 3.5 × 10^6 ^metric tonnes and USD 7 × 10^9 ^[18]. With the need to feed a growing population, an interest in healthy foods and the collapse of wild fisheries stocks, aquaculture has acquired a great importance [19,20]. Intensification of fish cultivation has largely targeted selection of faster growth rates and better feed conversion ratios. Fish feeds rely heavily on wild caught fish meal and oils, which puts further pressure on fish stocks and are a source of eutrophying pollutants [19-21]. The development of cultivation methods and new strains with increased productivity but at the same time the ability to digest alternative sources from plant material are therefore desirable objectives of the industry. They should decrease the dependency on capture fisheries [22]. Other objectives are strains with improved resistance to pathogens and tolerance to stress [23]. However, and although classical selection methods have an important role to play, genomic technologies can improve the genetic and biological basis of traits and allow direct selection on the genotype [23].

Economic and resource management interests have led to increased research efforts to develop genomics resources for European sea bass [24,25], including a >12 × coverage BAC-library [26], hundreds of microsatellite [27] and SNP markers [28], ESTs (Passos et al., unpublished), a genetic linkage map [29,30] and a radiation hybrid map (Senger, Galibert et al., unpublished). The European sea bass nuclear DNA content has been estimated at 1.55-1.58 pg [31] approximately twice that of *T. rubripes *[32], which, despite of advances in sequencing technologies, remains a large financial and logistic hurdle.

With time strategies for full *de novo *sequencing of large eukaryote genomes have shifted from whole genome shotgun (WGS) Sanger sequencing of cloned genomic DNA [8] to a combination of mapped large insert clone and WGS sequencing [33,34]. Today, with the evolution of second generation sequencing technologies, the re-sequencing of eukaryote genomes by massive parallel WGS sequencing is feasible [35]. It is expected that second generation sequencing technologies and especially pyrosequencing, which has been shown to cut costs and speedup the *de novo *sequencing of microbial genomes [36] will further contribute to reducing costs and time to sequence large genomes of higher eukaryotes.

In a pilot study for sequencing the genome of Atlantic salmon (*Salmo salar*) by Quinn et al. [37] pyrosequencing was useful for the generation of a draft sequence of a megabase sized genomic region. It also turned out that repeat richness in eukaryote genomes is the major problem for *de novo *sequencing with second generation technologies. Sequence-repeats resulted in a large amount of gaps in the assembly as they could not be resolved with reads shorter than the repeat itself, even if paired-end tags were used to scaffold the assembled contigs. A "first map, then sequence" strategy improves this situation as large genomes can be split into smaller subunits, which is one argument for genome mapping with large insert clones. Moreover hybrid assembly of Sanger sequencing data and short read data benefits from both technologies, finding a good balance of cost and quality [38].

Here we describe a comparative BAC-map and low coverage draft of the European sea bass genome obtained by high-throughput Sanger-sequencing of BAC-libraries and whole genome shotgun plasmid libraries as well as the exploitation of the synteny between *D. labrax *and *G. aculeatus*. The dataset represents the first whole genome sequencing of a fish belonging to the order of Perciformes and of a cultivated fish species, and sets the basic conditions for complete genome sequencing by second generation techniques in the near future.

## Results

### BAC-end sequencing

After quality clipping (> 300 Q20 bases) and removal of vector contamination, 102,690 BAC-end sequences (ES) with an average read length of 670 bp remained for analysis (sequences were submitted to EMBL nucleotide database [EMBL:FN436279 - EMBL:FN538968]. For a total of 44,836 BAC-clones, paired end sequences (BAC-ES) were determined, while for 13,018 BAC-clones only one ES was obtained. The estimated genome size of *D. labrax *based on diploid nuclear DNA content [31] is approximately 763 Mbp, suggesting that with an average insert size of 164 kbp per BAC, the genome coverage of paired end-sequenced BACs is about 9.6 fold. Clones that were sequenced only from one side sum up to an additional 2.8 fold genome coverage (see Table 1).

**Table 1:** BAC end sequencing results.

	both ends	only forward	only reverse	total
**BACs with good seq.**	44,836	7,235	5,783	57,854

**sequencing coverage [bp]**	60,073,046	4,846,870	3,874,146	68,794,062

**physical coverage [x-fold]**	9.6	1.6	1.2	12.4

The physical coverage was calculated by conservatively estimating a genome size of 763 Mbp for sea bass and an average BAC insert size of 164 kbp.

For comparative mapping, a subset of 10,000 BAC-ES was chosen to perform BLASTN searches with an e-value cut-off of 1e^-5 ^against the genomes of *D. rerio *[10], *T. nigroviridis *[7]], *O. latipes *[9] and *G. aculeatus *http://www.ensembl.org. The genomic sequence of *T. rubripes *[8]] was not used as the genome assembly has not been assigned to chromosomes. The highest number of matches was obtained against *G. aculeatus *(4,359 ES matches), followed by *O. latipes *(2,702), *T. nigroviridis *(2,536) and *D*. rerio (1,128). The results reflect known phylogenies, with *D. rerio *(superorder *Ostariophysii*) distantly related to the other candidates (all from the superorder *Acanthopterygii*) [39].

### Whole genome shotgun sequencing

Sequencing of whole genome shotgun libraries yielded >2 × 10^6 ^reads with an average Q20 read length of 673 bp comprising ~1.4 Gbp and approximately twofold coverage of the *D. labrax *genome. Assembly of WGS-reads and BAC-ES yielded 273,453 contigs and 217,926 singlets covering ~580 Mbp. The N50 contig size was 2,891 bp and the largest contig was 15,629 bp. A part of this dataset, namely 36,166 contigs were useful to anchor additional BAC-ES to the stickleback genome (see below). These contigs have been submitted to EMBL nucleotide database [EMBL:CABK01000001 - EMBL:CABK01036166].

### Comparative mapping

**Figure 1:**
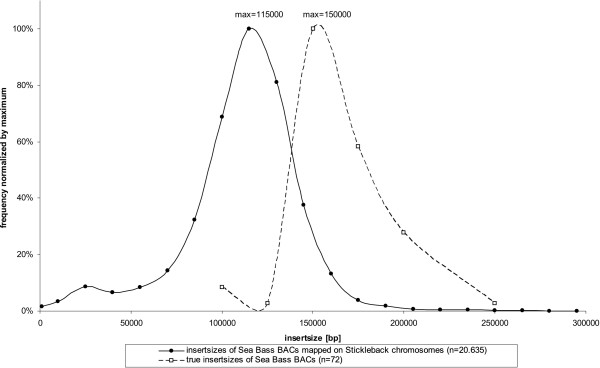
**Comparison of BAC insert size frequency distributions**. Insert sizes calculated by sea bass BAC-ES, consistently mapped to stickleback chromosomes, show a shift to lower insert sizes compared to the observed insert size of the library published by Whitaker et al. [26]. This observation can be explained by genome size evolution in teleosts. As the genome of sea bass is known to be larger than the stickleback genome the lower insert size of mapped BACs reflects the average size difference of orthologous loci. By dividing the insert sizes at the maxima of the distributions, it can be concluded that the sea bass genome is about 1.3 fold larger than the stickleback genome.

The whole BAC-ES dataset was aligned with the fully assembled stickleback genome. Further sorting and screening yielded 25,845 BAC-ES where only one end was sequenced or matched the stickleback genome and 13,996 BACs matched both ends to the same chromosome in stickleback. 18,013 BACs were matching weakly and were excluded (mostly due to repetitive motifs or possible chimeric BACs). BACs with both ES aligned were essential for comparative mapping and could be subdivided into 12,076 BACs with correct orientation and distance of aligned ES and 1,920 BACs not matching these consistency criteria due to possible rearrangements, miss-alignments or assembly failures in the stickleback genome. Plotting the frequency distribution of insert size of consistently mapped BACs resulted in a Gaussian-like distribution with a maximum at 115 kbp. This reflects a compression of the stickleback genome compared to the *D. labrax *genome, as the average insert size published for *D. labrax *is about 164 kbp [26] (see Fig. 1).

*D. labrax *BACs that were consistently positioned in the stickleback genome were used to calculate a minimal tiling path of overlapping BAC-clones resulting in 816 BAC-contigs that cover 78.1% of the 400.8 Mbp stickleback chromosomes and consisted of 3,629 BACs. The minimal tiling path of the largest BAC-contig comprised 52 BACs and covered 5.03 Mbp on *G. aculeatus *linkagegroup VI. N50 BAC-contig size was 0.53 Mbp. In the chromosomal regions covered by comparatively mapped BACs 77.5% of annotated genes assigned to stickleback chromosomes can be found (see Table 2, Table 3 and Table 4/values in brackets).

**Table 2:** Mapping statistics for sea bass BAC-ES comparatively mapped to the stickleback chromosomes I to VII.

A	stickleback chromosome	ALL CHR	CHR I	CHR II	CHR III	CHR IV	CHR V	CHR VI	CHR VII
	
	stickleback chromosome size [bp]	400,788,495	28,185,914	23,295,652	16,798,506	32,632,948	12,251,397	17,083,675	27,937,443
	
	genetic linkage group sea bass	-	LG13	LG5	LG10	LG2/?	LG1	LG11	LG3/LG14
**B**	**sea bass BAC-contigs**	588 (816)	34 (58)	28 (44)	24 (29)	42 (62)	16 (28)	24 (36)	43 (65)
	
	**largest BAC-contig [bp]**	8,860,934 (5,034,967)	6,029,661 (4,511,889)	4,235,816 (2,100,554)	3,575,021 (2,455,450)	7,720,774 (3,886,881)	1,931,357 (1,573,884)	8,860,934 (5,034,967)	4,327,197 (2,521,767)
	
	**% of chr. covered by BAC-contigs**	87.0% (78.1%)	84.8% (75.6%)	90.0% (83.6%)	85.5% (76.0%)	86.6% (76.2%)	88.5% (83.4%)	85.3% (77.5%)	89.3% (78.9%)
	
	**number of BACs in min. tiling path**	3,929 (3,629)	271 (257)	227 (221)	161 (144)	313 (283)	121 (118)	162 (147)	281 (266)
	
	**paired end aligned consistent BACs**	20,635 (12,076)	1,465 (802)	1,254 (772)	874 (510)	1,593 (883)	731 (418)	993 (678)	1,486 (849)
	
	**one end aligned BACs**	24,940 (25,845)	1,759 (1,781)	1,399 (1,453)	1,199 (1,196)	2,012 (2,111)	784 (839)	1,052 (1,087)	1,855 (1,940)

C	**inconsistently aligned same chr.**	1,487 (1,920)	98 (128)	77 (100)	93 (128)	111 (152)	57 (80)	64 (96)	97 (132)
	
	**potential intra-chr. rearrangements between stickleback and sea bass**	214	12	8	17	10	6	9	14
	
	**intra-chr. rearrangements also found between medaka and stickleback**	139	9	5	17	8	2	1	10
	
	**BAC-contigs with same neighbour in medaka as in stickleback**	149	7	4	4	16	4	7	7

**D**	**total annotated genes in ensembl**	19,045	1,253	853	923	1,317	733	749	1,311
	
	**% genes covered by BAC-contigs**	85.4% (77.5%)	80.5% (73.9%)	85.2% (78.8%)	86.5% (79.2%)	88.3% (79.3%)	88.4% (83.2%)	78.2% (72.0%)	86.7% (76.1%)

Rows (**A**) show a summary of the 21 reference chromosomes (stickleback assembly: BROAD S1, Feb 2006) and the known corresponding genetic linkage groups of sea bass according to Chistiakov et al. [30]. Rows (**B**) summarize consistently mapped BAC-ES, BAC-contigs and reference genome/chromosome coverage of the minimal tiling path. Rows (**C**) focus on inconsistently mapped BAC-ES, which where further analysed for potential intra-chromosomal rearrangements and compared to the medaka genome as a second reference genome. Rows (**D**) display the number of annotated genes in stickleback and the percentage of them covered by the sea bass BAC-map. Values in brackets "()" show results for a comparative map that was built by using only BAC-ES data, while all other values represent results of improved mapping using BAC-ES together with whole genome shotgun data.

**Table 3:** Mapping statistics for sea bass BAC-ES comparatively mapped to the stickleback chromosomes VIII to XIV.

A	stickleback chromosome	CHR VIII	CHR IX	CHR X	CHR XI	CHR XII	CHR XIII	CHR XIV
	
	stickleback chromosome size [bp]	19,368,704	20,249,479	15,657,440	16,706,052	18,401,067	20,083,130	15,246,461
	
	genetic linkage group sea bass	LG4	LG7	LG9	LG8	?	LG20	LG19
**B**	**sea bass BAC-contigs**	30 (44)	37 (45)	31 (37)	30 (27)	27 (35)	26 (38)	18 (33)
	
	**largest BAC-contig [bp]**	4,711,769 (3,129,353)	3,058,355 (1,487,429)	1,368,409 (1,368,348)	4,014,839 (3,993,770)	4,710,925 (2,950,196)	3,478,171 (3,182,188)	3,545,956 (1,757,446)
	
	**% of chr. covered by BAC-contigs**	85.8% (77.0%)	82.9% (69.7%)	84.2% (75.4%)	83.3% (74.4%)	88.5% (78.1%)	90.6% (81.8%)	88.0% (77.5%)
	
	**number of BACs in min. tiling path**	188 (177)	195 (174)	152 (140)	155 (147)	177 (160)	194 (176)	150 (142)
	
	**paired end aligned consistent BACs**	869 (517)	855 (448)	712 (410)	816 (494)	902 (559)	1,121 (678)	792 (414)
	
	**one end aligned BACs**	1,002 (1,190)	1,190 (1,168)	897 (879)	1,101 (1,089)	1,211 (1,364)	1,194 (1,266)	931 (1,017)

**C**	**inconsistently aligned same chr.**	110 (112)	84 (90)	90 (118)	81 (86)	58 (90)	42 (80)	59 (62)
	
	**potential intra-chr. rearrangements between stickleback and sea bass**	10	12	16	13	12	9	6
	
	**intra-chr. rearrangements also found between medaka and stickleback**	5	9	13	7	9	3	2
	
	**BAC-contigs with same neighbour in medaka as in stickleback**	15	12	6	8	6	7	9

**D**	**total annotated genes in ensembl**	876	1,009	802	1,050	1,000	970	738
	
	**% genes covered by BAC-contigs**	83.4% (75.9%)	80.0% (70.5%)	81.2% (70.7%)	83.5% (75.3%)	86.8% (76.5%)	89.6% (81.6%)	85.2% (74.9%)

Rows (**A**) show a summary of the 21 reference chromosomes (stickleback assembly: BROAD S1, Feb 2006) and the known corresponding genetic linkage groups of sea bass according to Chistiakov et al. [30]. Rows (**B**) summarize consistently mapped BAC-ES, BAC-contigs and reference genome/chromosome coverage of the minimal tiling path. Rows (**C**) focus on inconsistently mapped BAC-ES, which where further analysed for potential intra-chromosomal rearrangements and compared to the medaka genome as a second reference genome. Rows (**D**) display the number of annotated genes in stickleback and the percentage of them covered by the sea bass BAC-map. Values in brackets "()" show results for a comparative map that was built by using only BAC-ES data, while all other values represent results of improved mapping using BAC-ES together with whole genome shotgun data.

**Table 4:** Mapping statistics for sea bass BAC-ES comparatively mapped to the stickleback chromosomes XV to XXI.

A	stickleback chromosome	CHR XV	CHR XVI	CHRXVII	CHRXVIII	CHRXIX	CHR XX	CHR XXI
	
	stickleback chromosome size [bp]	16,198,764	18,115,788	14,603,141	16,282,716	20,240,660	19,732,071	11,717,487
	
	genetic linkage group sea bass	LG13	LG15	LG1?	LG17	LG6	LG16/18?	LG18?
**B**	**sea bass BAC-contigs**	20 (29)	24 (38)	30 (30)	35 (43)	38 (43)	22 (35)	9 (17)
	
	**largest BAC-contig [bp]**	3,426,120 (1,723,928)	6,616,511 (2,444,746)	3,516,936 (2,019,946)	1,934,982 (1,280,086)	2,450,075 (2,125,025)	7,220,725 (2,296,377)	4,139,228 (2,305,187)
	
	**% of chr. covered by BAC-contigs**	89.3% (82.5%)	88.4% (79.0%)	88.5% (78.7%)	83.6% (70.6%)	86.6% (78.2%)	90.3% (82.8%)	87.4% (83.3%)
	
	**number of BACs in min. tiling path**	156 (150)	182 (167)	163 (138)	170 (143)	206 (185)	194 (178)	111 (116)
	
	**paired end aligned consistent BACs**	969 (619)	916 (533)	784 (459)	783 (373)	1,004 (628)	1,077 (639)	639 (393)
	
	**one end aligned BACs**	1,017 (1,057)	1,163 (1,162)	1,110 (1,145)	989 (1,040)	1,204 (1,187)	1,178 (1,183)	693 (691)

**C**	**inconsistently aligned same chr.**	73 (96)	47 (74)	37 (34)	68 (78)	104 (128)	30 (46)	7 (10)
	
	**potential intra-chr. rearrangements between stickleback and sea bass**	10	8	6	12	14	8	2
	
	**intra-chr. rearrangements also found between medaka and stickleback**	9	4	4	6	8	6	2
	
	**BAC-contigs with same neighbour in medaka as in stickleback**	3	7	4	9	7	3	4

**D**	**total annotated genes in ensembl**	779	799	698	761	1,037	927	460
	
	**% genes covered by BAC-contigs**	87.9% (84.1%)	88.5% (80.4%)	85.5% (77.4%)	82.7% (69.5%)	82.5% (75.5%)	89.5% (85.1%)	93.9% (86.7%)

Rows (**A**) show a summary of the 21 reference chromosomes (stickleback assembly: BROAD S1, Feb 2006) and the known corresponding genetic linkage groups of sea bass according to Chistiakov et al. [30]. Rows (**B**) summarize consistently mapped BAC-ES, BAC-contigs and reference genome/chromosome coverage of the minimal tiling path. Rows (**C**) focus on inconsistently mapped BAC-ES, which where further analysed for potential intra-chromosomal rearrangements and compared to the medaka genome as a second reference genome. Rows (**D**) display the number of annotated genes in stickleback and the percentage of them covered by the sea bass BAC-map. Values in brackets "()" show results for a comparative map that was built by using only BAC-ES data, while all other values represent results of improved mapping using BAC-ES together with whole genome shotgun data.

**Figure 2:**
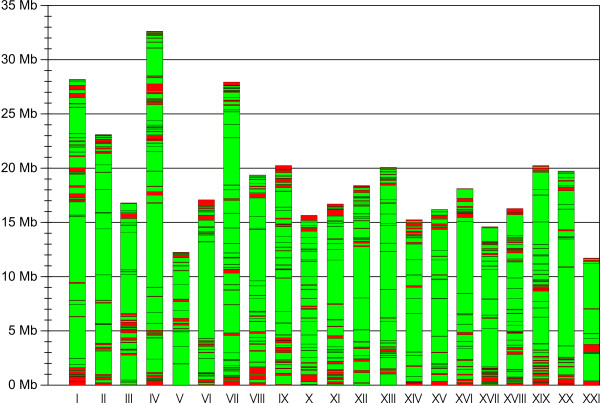
**Visualization of comparative sea bass BAC map on 21 stickleback chromosomes**. Green regions represent BAC-contigs, which are covered on average 5.25 fold by consistently mapped BAC-clones. In total 87.0% of the stickleback chromosomes and 85.4% of annotated genes are covered by the BAC-contigs. Regions with weak mapping results are shown in red and mainly due to repetitive regions like centromeric or telomeric regions, where it is hard to consistently align both BAC-ES. Nevertheless BACs having good matches with one end may be found in these regions. Most of the stickleback chromosomes can be assigned to sea bass genetic linkage groups (see Table 2A, Table 3A and Table 4A).

Comparative mapping was improved by aligning BAC-ES containing contigs from the WGS and BAC-ES assembly to stickleback chromosomes. This strategy yielded 20,635 BACs matching consistency criteria, an improvement of about 71% compared to comparative mapping using only BAC-ES data. The re-calculated minimal tiling path reduced total contig number to 588 and N50 contig number to 83 while increasing N50 BAC-contig size to 1.2 Mbp and coverage of stickleback chromosomes to 87% (see Table 2, Table 3, Table 4 and Fig. 2, a complete list of ordered paired-end aligned BAC clones on stickleback chromosomes may be downloaded [Additional file 1]).

**Figure 3:**
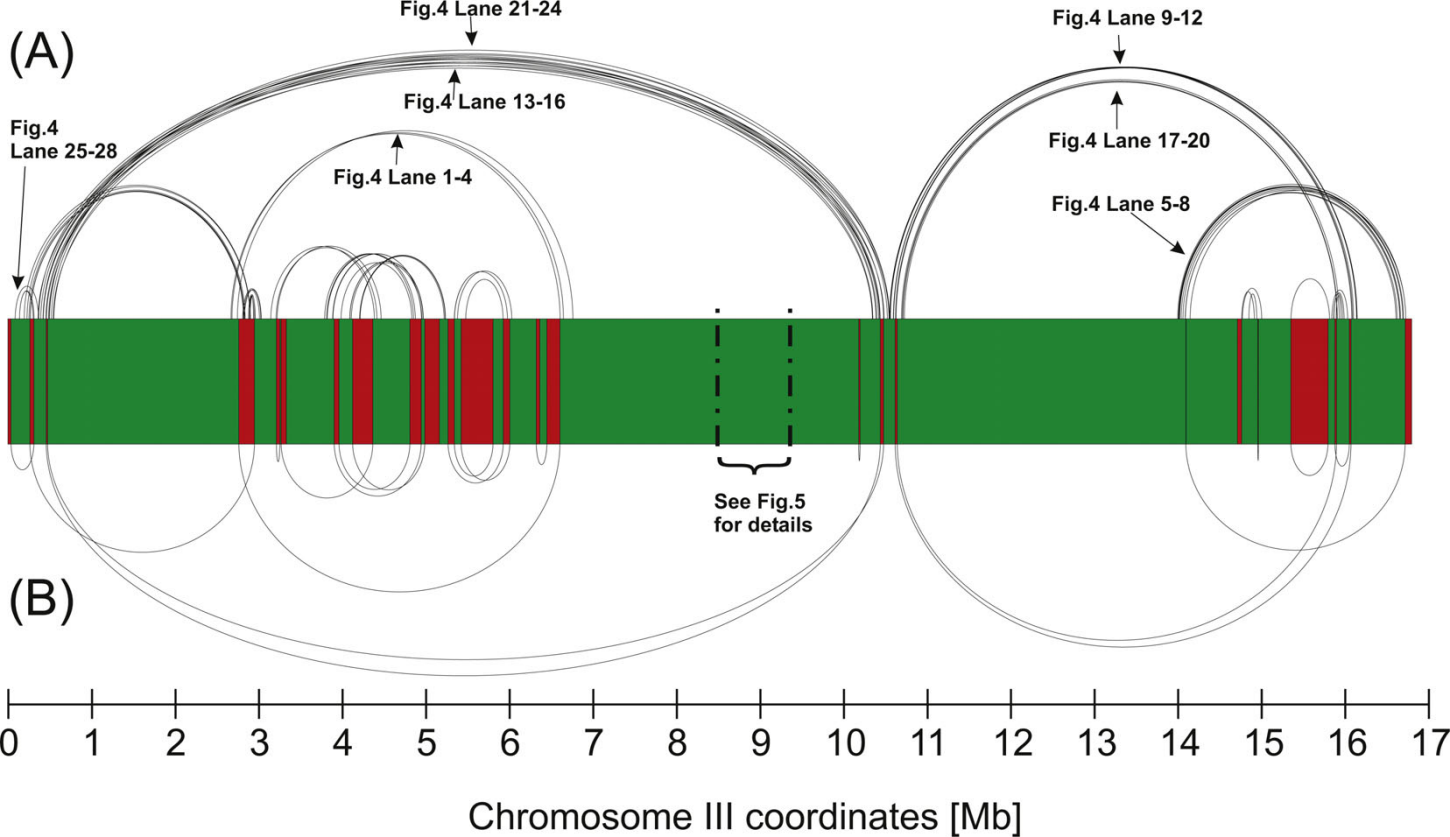
**A closer look at potential intra-chromosomal rearrangements**. (**A**) For each stickleback chromosome, sea bass BAC clones can be determined whose BAC-ES pairs are inconsistently aligned in terms of distance or alignment orientation. If these pairs are incorporated in the mapping visualization as black lines, it can be found that edges of BAC-contigs are connected by these clones. It is very likely that these clones represent candidates that span intra-chromosomal rearrangements between stickleback and sea bass. (**B**) To increase significance of rearrangements and exclude rearrangements that were proposed due to chimeric BAC clones, the position of edges of BAC contigs was cross-checked on medaka chromosomes. For chromosome III we found that most of the rearrangements between sea bass and stickleback could be confirmed by comparison with medaka as a second reference genome. In total 214 potential rearrangements between sea bass and stickleback chromosomes could be pinpointed; about 65% of these were confirmed by comparison with medaka (see Table 2C, Table 3C and Table 4C). For visualizations of all chromosomes see supplemental data section. Rearrangements that were evaluated by PCR are labelled with the corresponding lanes in Fig. 4. For a detailed view of BACs ordered between 8.47 Mbp and 9.47 Mbp see Fig. 5.

Moreover the higher coverage with BACs enabled the identification of potential intra-chromosomal rearrangements between sea bass and stickleback (or failures in the stickleback assembly). A number of 214 potential chromosomal breakpoints spanned by BAC-clones were identified [Additional file 2]. To check if rearrangements were artefacts, the order of calculated BAC-contigs was cross-checked by alignment to the medaka genome (see Fig. 3). In total 139 cases (65%), had a neighbouring position at that site and thus confirmed the consistency of the identified BAC-clone on the second reference genome.

**Figure 4:**
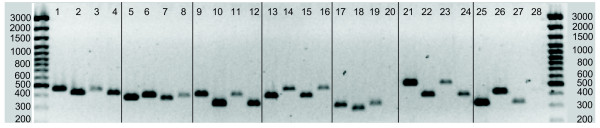
**Evaluation of seven potential rearrangements between stickleback chr III and the corresponding sea bass linkage group 10 by means of PCR**. Primers were designed on sea bass BAC-ES representing the ends of BAC contigs that seem to be connected by a rearrangement spanning BAC-clone. PCR was performed on the connecting BAC to proof the overlap with both BAC contigs and on genomic DNA to check that the PCR product was a unique marker in the sea bass genome. The amplified markers are shown above, for each rearrangement the first and second lane represents the markers amplified on the rearrangement spanning BAC, the third and fourth lane shows the same markers amplified on genomic sea bass DNA. **Lane 1-4**: bassbac140-o20/stickleback chr III 6.6 Mbp <> 2.66 Mbp. **Lane 5-8**: bassbac-137j6/stickleback chr III 14.06 Mbp <> 16.65 Mbp. **Lane 9-12**: bassbac-1g24/stickleback chr III 16.14 Mbp <> 10.6 Mbp. **Lane 13-16**: bassbac-38h23/stickleback chr III 0.5 Mbp <> 10.55 Mbp. **Lane 17-20**: bassbac-52b18/stickleback chr III 15.89 Mbp <> 10.64 Mbp. **Lane 21-24**: bassbac42b12/stickleback chr III 0.457 Mbp <> 10.44 Mbp. **Lane 25-28**: bassbac49h3/stickleback chr III 0.035 Mbp <> 0.311 Mbp. Each of the seven BACs had overlaps with the two BAC-contigs predicted by the comparative mapping approach. 12 out of 14 markers were unique in the sea bass genome. 2 markers (Lane 20 and 28) could not be amplified using genomic DNA as a template.

Fig. 4 shows PCR results that support the bioinformatic data on rearrangements between sea bass and stickleback. All of the seven rearrangements that were checked by PCR have been confirmed. For each of these rearrangements we found at least 2 BAC clones that gave positive results in the PCR, the average number of BAC clones spanning a rearrangement was 4.6 and the maximum number was 7 clones.

**Figure 5:**
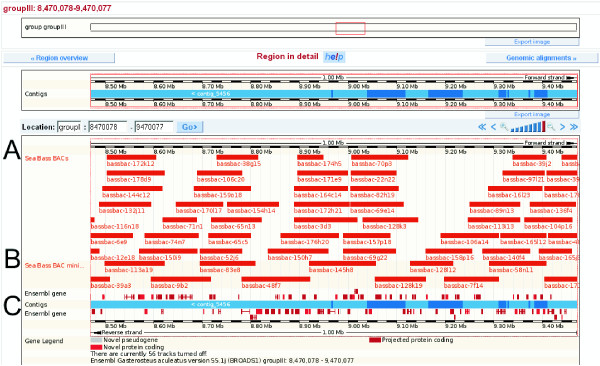
**BAC clones in the Ensembl browser**. The mapped BAC clones (**A**, **B**) may be browsed alongside the stickleback chromosomes by the Ensembl genome browser (http://www.ensembl.org/Gasterosteus_aculeatus login: kuhl@molgen.mpg.de password: BASSBACMAP2009). This way sea bass BACs can be easily assigned to annotated stickleback genes (**C**). BACs forming the minimal tiling path are displayed separately (**B**).

The consistently mapped BACs were also uploaded to the Ensembl genome browser and may be viewed in a user friendly format alongside the annotated stickleback chromosomes (see Fig. 5)

## Discussion

Recently BAC-end sequencing has been a tool for scaffolding large eukaryotic genome assemblies and thus became important in the final phase of sequencing projects. Today as the number of eukaryotic genomes in the databases is steadily increasing, comparative mapping approaches will change that picture. In the case of *Dicentrarchus labrax *BAC-end sequencing started before a whole genome project was even planned and enabled a fast and cost-effective mapping of the genome.

### Comparative mapping compared to other mapping strategies

Since publication of the first BAC-vector [40] several strategies for the construction of physical genome maps from BAC-libraries have been published. Among these methods BAC-filter hybridization [41], BAC-fingerprinting [42] and PCR screening [43] have been applied most frequently. Comparative mapping approaches are likely to replace these methods because many genomes of higher eukaryotes have been published. Comparative maps are built by aligning paired end sequences of large insert clones (e.g. BACs) to a reference genome and thus detecting possible overlaps of clones that subsequently can be combined into contigs. This strategy has been successfully applied to closely related organisms such as chimpanzee and human [44] and also to more distantly related organisms like cattle and human [45]. Comparative mapping has some advantages for automated analysis over the methods mentioned above, as established pipelines for high-throughput sequencing and bioinformatics can be used.

### BAC end sequencing results

Sanger sequencing of BAC-ends remains restricted to a 96 well format in many sequencing centers, because of low template yields and large amounts of template used for the sequencing reactions. Thus the successful development of an automated DNA purification process to purify BAC-DNA from 384 well plates was a crucial step to enable the comparative genome mapping of *D. labrax*. With an average read length of 650 bp on 36 cm and 750 bp on 50 cm capillaries the read length of BAC-end sequences was substantially higher than reported in comparable projects [45]. Failed reactions were less than 11%.

### Reference genomes

Besides read quality, the choice of a suitable reference genome is influencing mapping success and quality. Several sequenced genomes of model teleosts are available (e.g. *D. rerio*, *T. nigroviridis*, *T. rubripes*, *O. latipes *and *G. aculeatus*). With the highest number of mappable reads, the stickleback genome sequences shared the highest homology to *D. labrax*, making it the genome of choice for a comparative approach. The stickleback and the European sea bass belong to the superorder Percomorpha, and the evolutionary related orders of Gasterosteiformes and Perciformes, respectively [46]. Additional beneficial features of the stickleback genome sequence is the high sequencing coverage (~12 fold) and the mapping of most scaffolds to chromosomes.

### The comparative map

After BAC-ES data for sea bass became available, a first comparative BAC-map was built. Results were already usable to render megabase sized contigs and to screen for BACs covering genes of interest. Subsequently with a WGS dataset of the sea bass genome available, BAC-ES sequences and WGS data were combined by assembly. If a BAC-ES alone could not be matched with the reference earlier on, the length extension of the BAC-ES by aligned WGS reads now increased the probability to find matches with good alignments to the reference genome. Final mapping (Fig. 2) shows in green that most of stickleback chromosomes are covered by *D. labrax *BACs with consistent orientation and distance (87% of reference genome), while red regions have a weak mapping, where no or only one BAC-end sequence could be matched. These regions may be either due to highly repetitive fragments, gaps and/or failures in the assembly of the sticklebackgenomic sequence or regions that are underrepresented in the BAC-library. It is obvious that especially centromeric and telomeric regions, known for the problems mentioned above, account for weakly mapped regions.

### Calculating the genome size of *D. labrax*

When comparing the insert size distribution of consistently mapped BACs on the reference genome with the published insert size distribution of the *D. labrax *BAC-library (Fig. 1), a shift to lower insert sizes is observed. An explanation for this may be found in the evolution of genome size. It has been shown that teleost genomes tend to accumulate most indels in intergenic or intronic regions leading towards large differences in genome size, while synteny of genes is conserved [47]. Thus one may conclude that the ratio of the maxima in the insert size distributions of BAC-clones equals the ratio of genome sizes. From this calculation one may conclude that the *D. labrax *genome is about 1.3 fold larger than the 460 Mbp of the *G. aculeatus *genome. The calculated haploid genome size of 600 Mbp is smaller than the estimated haploid genome size of 763 Mbp derived from flow cytometric measurements of diploid nuclear DNA content [31]. A smaller genome size is also suggested by the first assembly of our twofold coverage WGS dataset (see WGS sequencing results). Nevertheless, genome size estimates from sequencing may be biased towards the euchromatic portion of the genomes and different results of the methods may be explained by underrepresentation or different size evolution of heterochromatic regions.

### Comparing the BAC and the linkage map of *D. labrax*

BAC contigs represented by green regions in Fig. 2 are considered blocks with a high level of synteny between *D. labrax *and *G. aculeatus*. Nevertheless it is questionable whether neighbouring BAC-contigs on the reference genome are really neighbours in the *D. labrax *genome or whether chromosomes have undergone extensive inter-chromosomal rearrangements during evolution. To decide either whether a rearrangement has taken place or the order of BAC-contigs is consistent in both genomes, it is helpful to compare results from the *D. labrax *genetic linkage map and the radiation hybrid map of the closely related sparid *Sparus aurata *(gilthead sea bream). Such comparisons with stickleback have been done by Chistiakov et al. [30] and Sarropoulou et al. [16] and showed synteny of complete chromosomes between these species. Chromosome identity and re-shuffling are common features among closely related organisms. The different chromosome number of *G. aculeatus *(n = 21) and *D. labrax *(n = 24) is a common feature between related taxa and can be explained by fusions/fissions of complete orthologous groups. Thus it is unlikely that BAC-contigs mapped to one *G. aculeatus *chromosome are not located on a single *D. labrax *chromosome. These results also allow assigning the comparatively mapped BAC-contigs to *D. labrax *linkage groups (Table 2A, Table 3A and Table 4A).

Comparison of the *D. labrax *linkage map with the *G. aculeatus *genome has suggested some intra-chromosomal rearrangements [30]. Due to the higher resolution of the comparative BAC-map, it is possible to pinpoint potential rearrangements by focussing on inconsistently mapped BACs that connect two BAC-contigs at their boundary regions. Since BAC-libraries are known to harbour some chimeric clones, the location of potentially neighbouring BAC-contigs was confirmed by cross-checking their position in the medaka genome. If BAC-contigs connected by a rearrangement spanning BAC were located next to each other in the medaka genome, a true rearrangement was considered (Fig. 3). In this way 139 BACs spanning rearrangements between the reference and *D. labrax *genome were identified. Seven rearrangements between chr III of stickleback and the corresponding sea bass linkage group 10 were also tested by means of PCR. All of them could be confirmed (Fig. 4).

### Applications

The main advantage of BAC-maps over other mapping methods, like genetic linkage maps or radiation hybrid maps, is the possibility to access defined portions of a genome for subsequent analysis by common methods of molecular genetics. As the comparative BAC-map covers about 85.4% of predicted *G. aculeatus *genes, it is now possible to easily access orthologous *D. labrax *genes by selecting a BAC-clone that covers the genomic region of interest. As proof of principle, we have successfully identified and shotgun sequenced 10 overlapping BAC-clones that cover a 1.3 Mbp genomic region on sea bass linkage group 5 (Negrisolo et al. in preparation). The BAC map was also used to analyze two clones that contain a novel immune-type receptor (NITR) gene cluster [48] and to sequence the fatty acid delta-6 desaturase gene in European sea bass (Santigosa et al. in preparation).

## Conclusions

The comparative approach enabled a fast and cost effective mapping of large genomic portions of the *D. labrax *genome; it was further refined by adding WGS data from the early stage sequencing project. Both, WGS- and BAC-end sequencing data now represent a solid basis for sequencing the complete genome in a "first map, then sequence" approach with second-generation techniques, such as pyrosequencing. The BAC-map allows splitting the genome into smaller BAC-pools (e.g. covering single chromosomes). This will facilitate the sequence assembly as short reads are a major problem of new sequencing technologies, when sequencing repeat-rich eukaryotic genomes.

The integration of linkage [30], radiation hybrid (Senger, Galibert, in preparation) and BAC-mapping (this study) of sea bass will certainly result in a high quality physical map of the genome. It sets the scene for quantifying polymorphisms and genomic architecture. These are powerful resources for quantitative trait loci mapping, which can be eventually applied in selective breeding using marker assisted selection or introgression [24]. There is also the possibility of genome wide association mapping, based on massive resequencing, to identify genomic regions affecting the phenotype [49,50]. Therefore it sets the basic conditions for research to improve the sustainability of sea bass aquaculture in the Mediterranean basin and (shell)fish aquaculture in general.

## Methods

### BAC end-sequencing

The *Dicentrarchus labrax *BAC-library constructed by Whitaker et al. [26] was obtained from the German resources center for genome research (RZPD, Berlin, Germany). The library comprises pCC1BAC-clones arrayed in 180 × 384 well microtiter plates. The total genome coverage of the library is >12 fold with an average insert size of 164 kbp per BAC-clone. For end sequencing, BAC-clones were inoculated in 2 × 384 deep well plates containing 190 μl of 2YT media and 12.5 mg/l chloramphenicol and cultivated for 18 h at 37°C with rigorous shaking at 1100 rpm in Titramax 1000 incubators (Heidolph Instruments). BAC-DNA was purified by an automated process that was developed at the MPI for molecular genetics. The process applies size selective precipitation in polyethylene-glycol 6000/2-propanol mixtures and a final washing step with ethanol 70% (v/v).

BAC-templates were end sequenced using ABI BigDyeV3.1 Terminator chemistry and T7 or SP6 primers. After post-sequencing cleanup by ethanol/NaAcetate precipitation, sequence analysis was performed on ABI3730 × l capillary sequencers with either 36 cm or 50 cm capillary arrays. Processing of raw sequencing data was done by the PHRED basecaller [51], quality clipping and vector-clipping by LUCY [52].

### Whole genome shotgun sequencing

For the construction of WGS plasmid libraries of *Dicentrarchus labrax*, we obtained genomic DNA isolated from the same specimen (male 57 originating from the Adriatic clade) that was used for BAC-library construction (kindly provided by A. Libertini, CNR, Venice, Italy through J. B. Taggart, University of Stirling, UK).

Genomic DNA was sheared by ultrasonic sound and size selected for fragment sizes of 0.9 - 1.5 kbp and 1.5 - 4 kbp. Fragments were polished by T4-DNA-polymerase/DNA-polymerase I (Klenow) and ligated with T4-DNA-Ligase into SmaI digested pUC19 sequencing vector. Competent *E. coli *DH10B cells (Invitrogen) were transformed by electroporation, plated on 22 × 22 cm agarplates (Nunc) containing LB media with 110 mg/l Ampicillin, X-GAL and IPTG. After 16 h of incubation at 37°C white colonies were arrayed into 384 well microtiter library plates by a picking robot (Q Bot, Genetix). These plates (media: LB+HMFM+Ampicillin) were again incubated for 16 h at 37°C and stored at -80°C. Plasmid DNA preparation was done as described for BAC-DNA with the difference that the final washing step with 70% (v/v) ethanol was not necessary and a single 384 deepwell microtiter plate filled with 190 μl of 2YT + 110 mg/l Ampicillin yielded enough template amounts for several sequencing reactions.

Sequencing, sequence analysis and sequence processing of plasmids was done as described above using ABI BigDyeV3.1 Terminator chemistry and M13(-40) or M13(-28) primers.

### Alignment of BAC-ES to reference genome

BAC-ES were aligned by BLAST [53,54] algorithms to genomic sequence of *G. aculeatus *(Assembly: BROAD S1, Feb 2006, http://www.ensembl.org). To minimize computational time BLAST searches were done incrementally beginning with stringent parameters (Megablast, word size 20, and nucleotide mismatch penalty -1). Results were filtered for alignments that matched with an e-value equal or lower than 10^-5^. Additionally, alignments were only submitted to further analysis, if the second best alignment resulted in an e-value that was at least 10^5^-fold larger than the e-value of the best alignment. Sequences with alignments not matching these criteria were extracted by notseq [55] and subsequently aligned by BLAST searches with lower stringency. Stringency in the following rounds was adjusted by choosing word sizes of 15, 11 and 7.

The number of BAC-ES with alignments meeting our criteria was further improved by adding sequences from whole genome shotgun sequencing of *D. labrax*. All sequences available were assembled by the Celera Assembler [56]. Contigs that contained BAC-ES were filtered and again aligned to the *G. aculeatus *genome as described above. Match coordinates of contigs on *G. aculeatus *chromosomes were corrected by position of the BAC-ES in the contigs and assigned to the corresponding BAC-ES.

### Calculating and visualizing the comparative map

Resulting BLAST-tables of both approaches were screened for BACs that were aligned with both ends. These BACs were further screened for matches to the same chromosome in *G. aculeatus *and then checked for consistent orientation and distance.

BACs that matched all consistency criteria were chosen for the calculation of a minimal tiling path. Starting with a first BAC-clone, BAC-contigs were constructed by choosing BACs that were overlapping and maximizing the contig in length. These analyses were done by common spreadsheet software and scripting language. BAC-contigs arranged on the 21 *G. aculeatus *chromosomes were visualized by passing coordinates to a vector graphics application (CorelDRAW version 11, Corel corp., Ottawa, Canada). To view the BAC map alongside the annotated stickleback genome the mapping coordinates were uploaded to the ensembl genome browser as a GFF formatted textfile.

### Dealing with possible rearrangements and checking them on a second reference genome

The subset of BAC-ES that aligned to the same chromosome but did not match consistency criteria could be due to intra-chromosomal rearrangements between stickleback and sea bass. These clones were visualized as black arcs on the stickleback chromosomes. If these arcs were starting not at the edge of contigs they were manually removed. To check if the rearrangement spanning BAC-ES have a consistent order in the medaka genome, we exploited stickleback and medaka synteny of orthologous genes. Using the biomart tool a table was prepared that showed the genes annotated to stickleback with their orthologous position in medaka. Furthermore the coordinates of contig starts and ends from the sea bass BAC-map were implemented in the table. In this way the position of sea bass contig starts and ends on medaka could be mapped to stickleback chromosome coordinates. BAC-contig edges that are located next to each other in medaka were subsequently visualized by arcs, in many cases confirming a connection between contigs that was also found before by non-consistent matching sea bass BAC-ES.

### Evaluation of several rearrangements by means of PCR

Seven potential rearrangements between stickleback chr III and the corresponding sea bass linkage group were checked by means of PCR. Primers for PCR were designed on BAC-ES representing the end of BAC-contigs that seem to be connected by a rearrangement spanning clone. Subsequently amplification of the chosen markers was carried out using the rearrangements spanning clones as templates. If both BAC-contig end markers can be amplified on a rearrangement spanning BAC, the overlap and therefore connection of the two BAC-contigs in sea bass is confirmed. Additionally markers were amplified on genomic DNA of sea bass to check that they are unique markers in the genome. The PCR was set up as 50 μl reactions. For amplification of BAC-templates we added 2 μl of overnight culture to the PCR, while amplification of genomic DNA was carried out by adding 2 μl DNA with a concentration of 45 ng/μl to the PCR. Composition of PCR was as follows: 0.3 μM for each primer, 300 μM dNTPs, 75 mM TRIS-HCl, pH 9, 20 mM (NH_4_)_2_SO_4_, 0.01% Tween 20, 2.5 mM MgCl_2_, 0.1 U/μl Taq-DNA-polymerase and 0.5 M Betaine. Thermocycler profile was: Step I: 5 min at 94°C. Step II: 30 s at 94°C. Step III: 30 s at 55°C. Step IV: 1 min at 72°C. Step V: 7 min at 72°C. Step VI: hold at 4°C. Steps II-IV were repeated 25 times. PCR products were analyzed on 1.5% agarose gels and stained with ethidium bromide.

## Authors' contributions

HK established protocols for automated BAC, plasmid purification and subsequent Sanger sequencing, assembled the derived sequencing data, built the comparative map of BAC clones and wrote the manuscript. AB provided tools for bioinformatic analyses. GW programmed robotics for the automation of the purification process. AVMC and FAMV helped writing the manuscript. RR provided robotics, sequencing technologies and corrected the manuscript. All authors read and approved the manuscript.

## Supplementary Material

Additional file 1 **BACs_mapped**. The file contains BAC clones mapped to stickleback chromosomes in a BLAST table format. Click here for file

Additional file 2 **rearrangements_all_chr**. The file contains the 21 stickleback chromosomes (page 1 = chr I; ....; page 21 = chr XXI), showing potential intra-chromosomal rearrangements between sea bass and stickleback (left) and similar rearrangements between medaka and stickleback (right). Click here for file
