# Supplementary figures and images for: The European sea bass Dicentrarchus labrax genome puzzle: comparative BAC-mapping and low coverage shotgun sequencing
Source: BMC Genomics. 2010 Jan 27;11:68. doi: 10.1186/1471-2164-11-68 (PMC2837037; doi:10.1186/1471-2164-11-68)

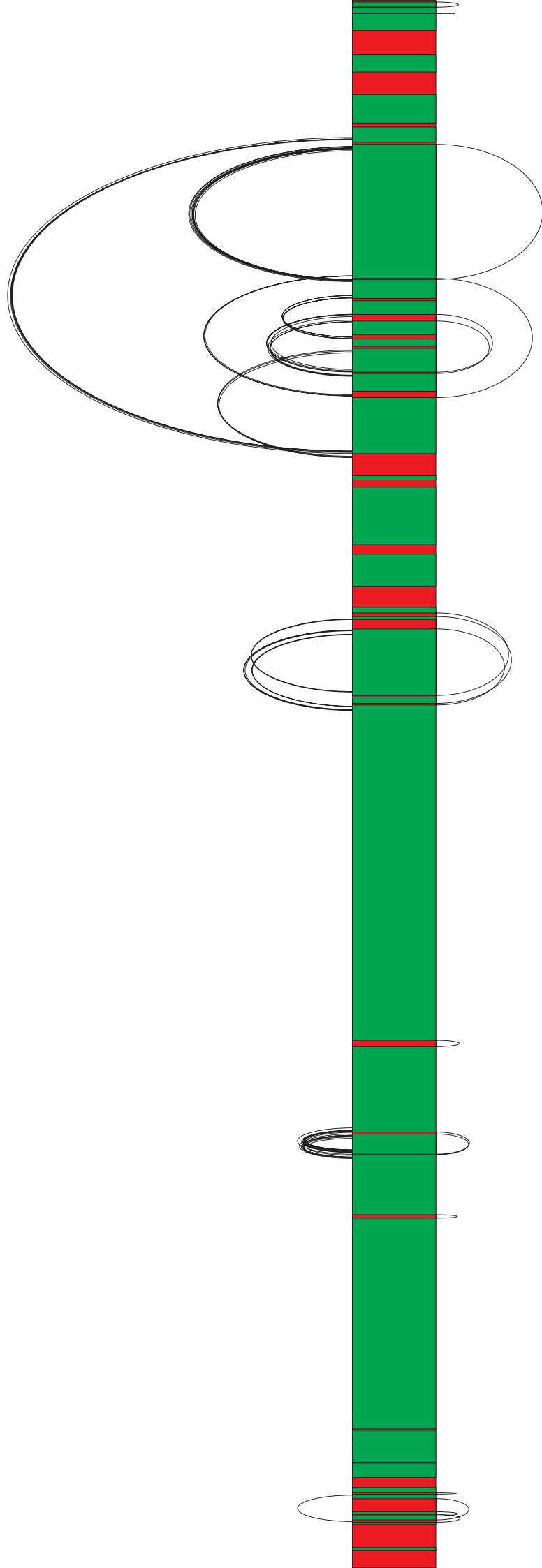

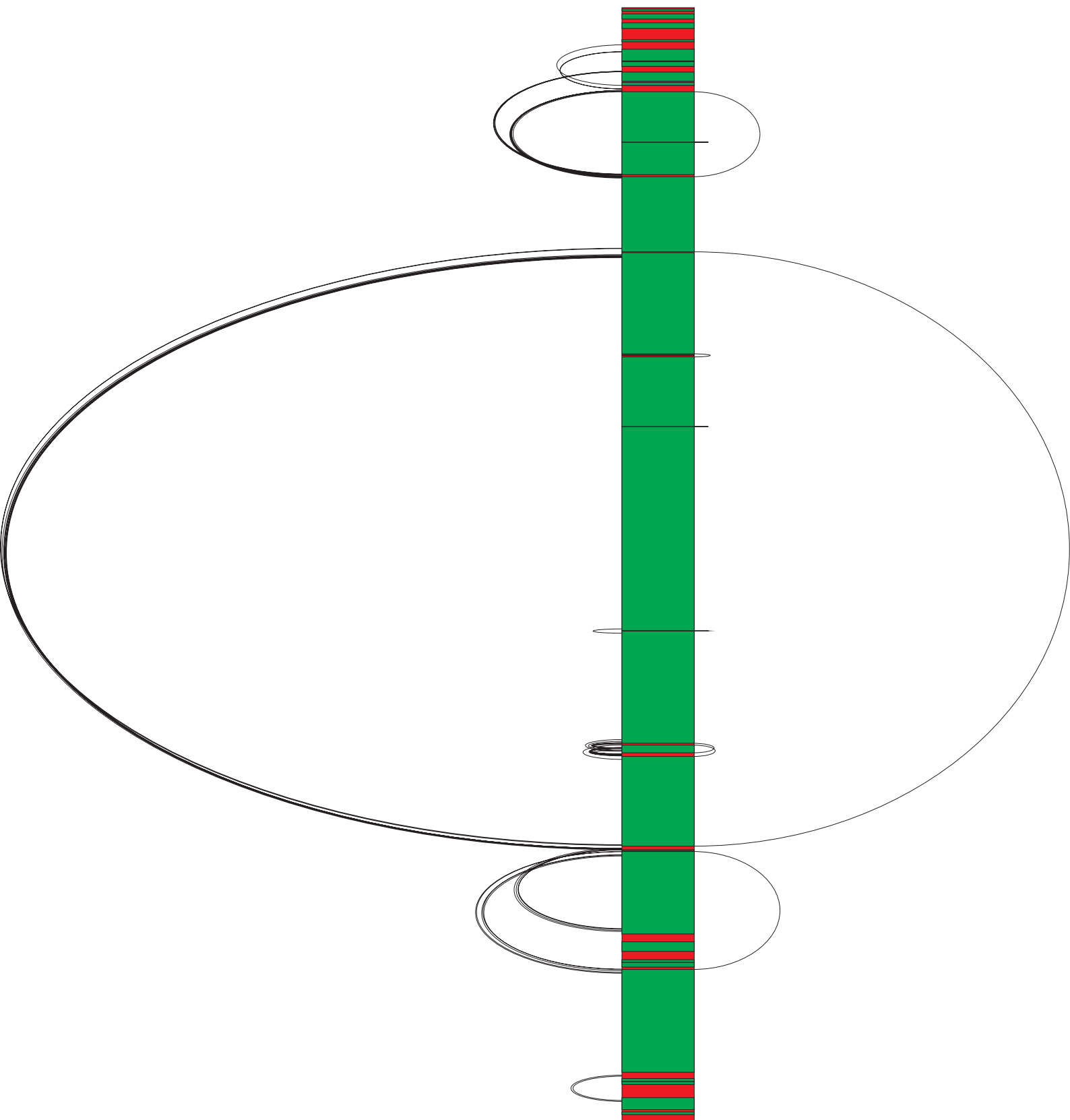

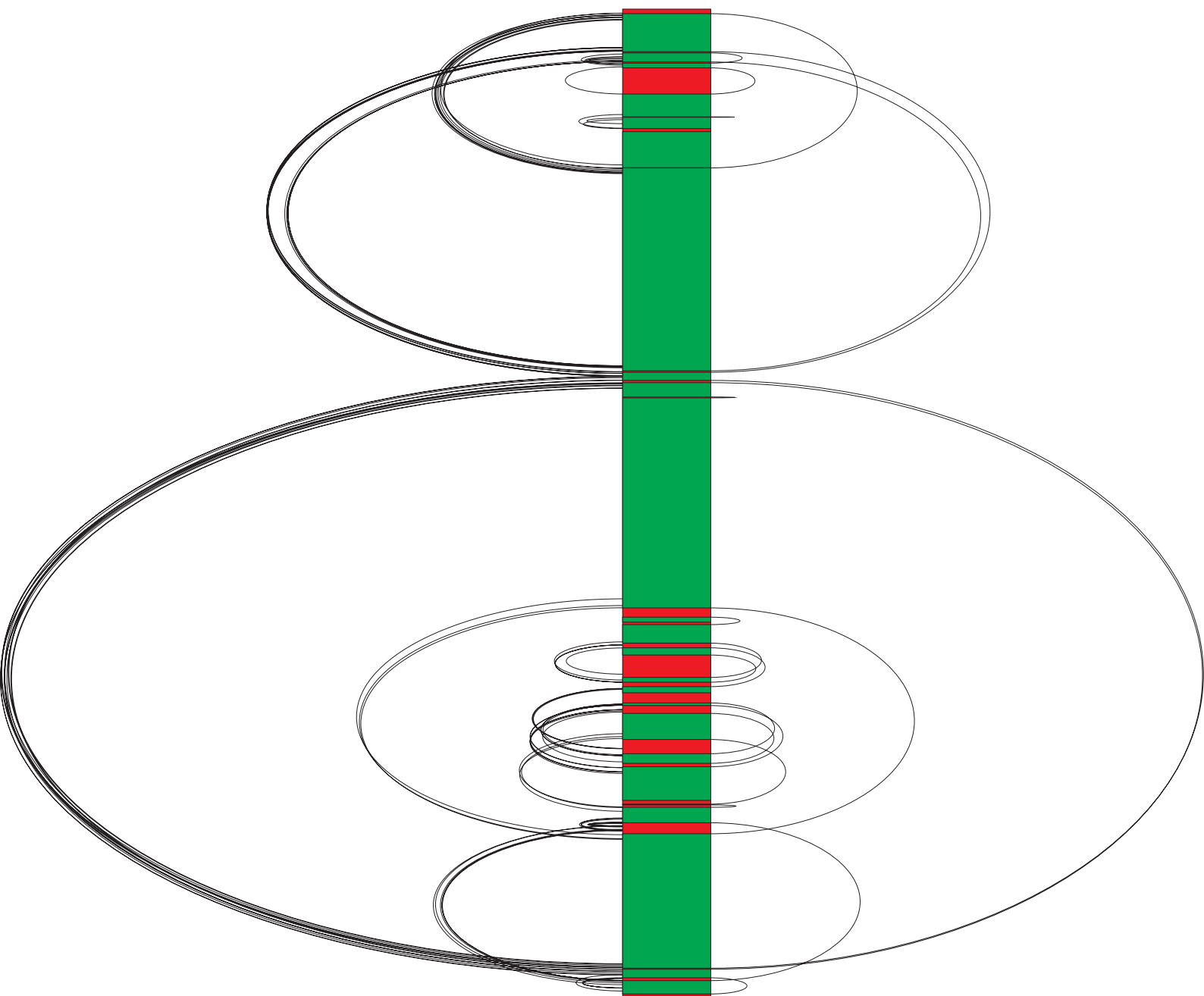

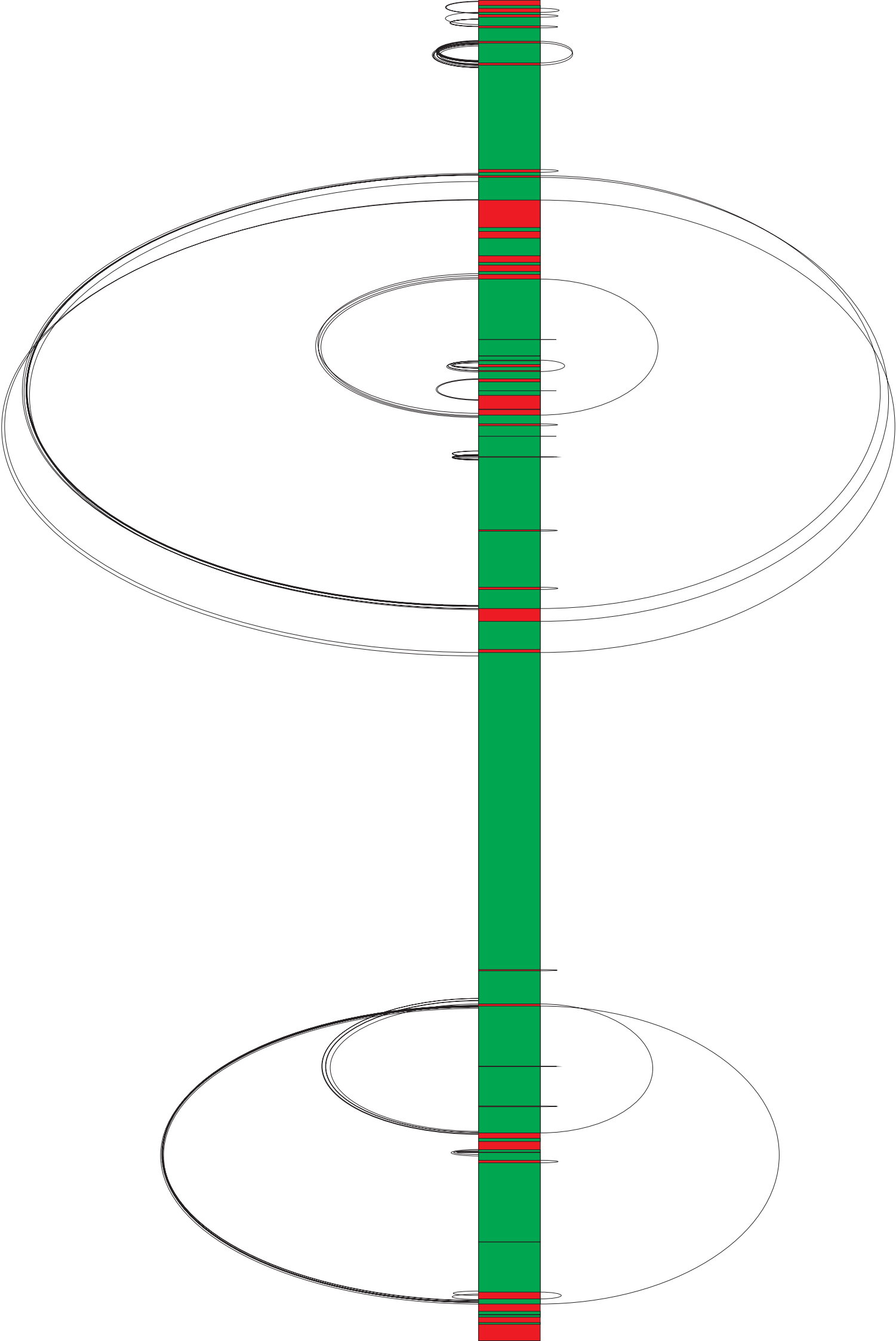

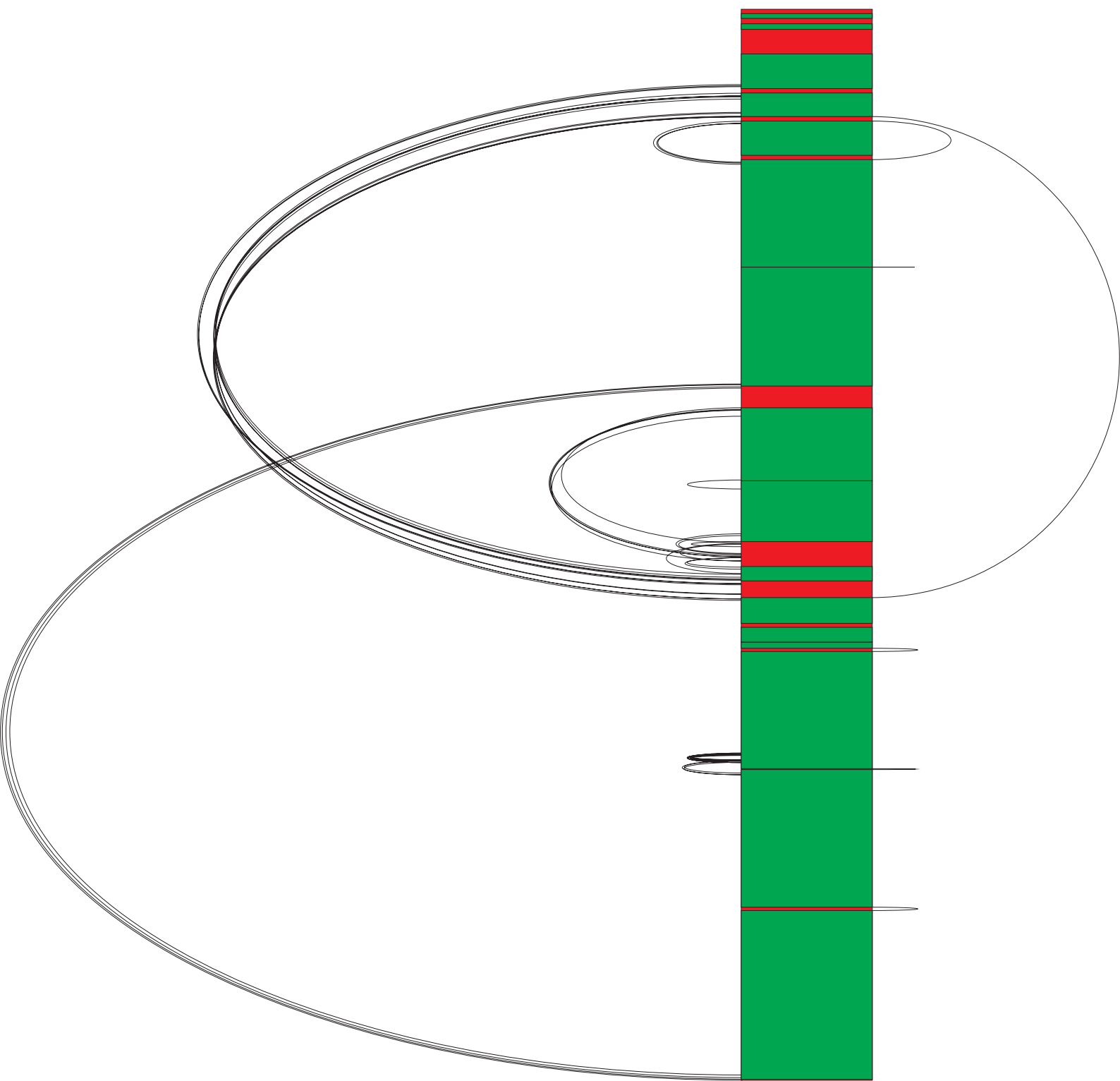

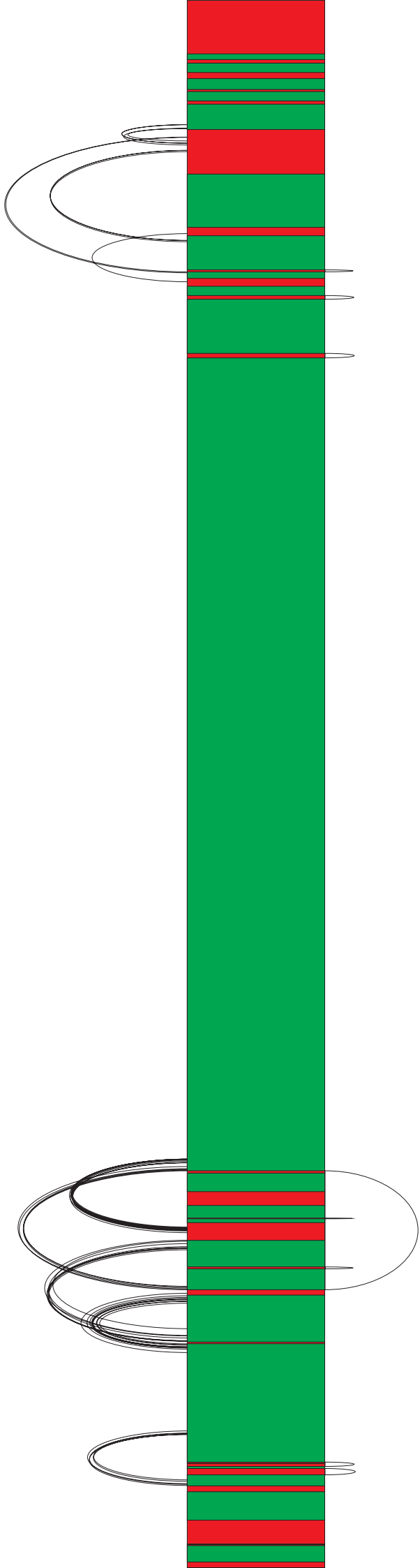

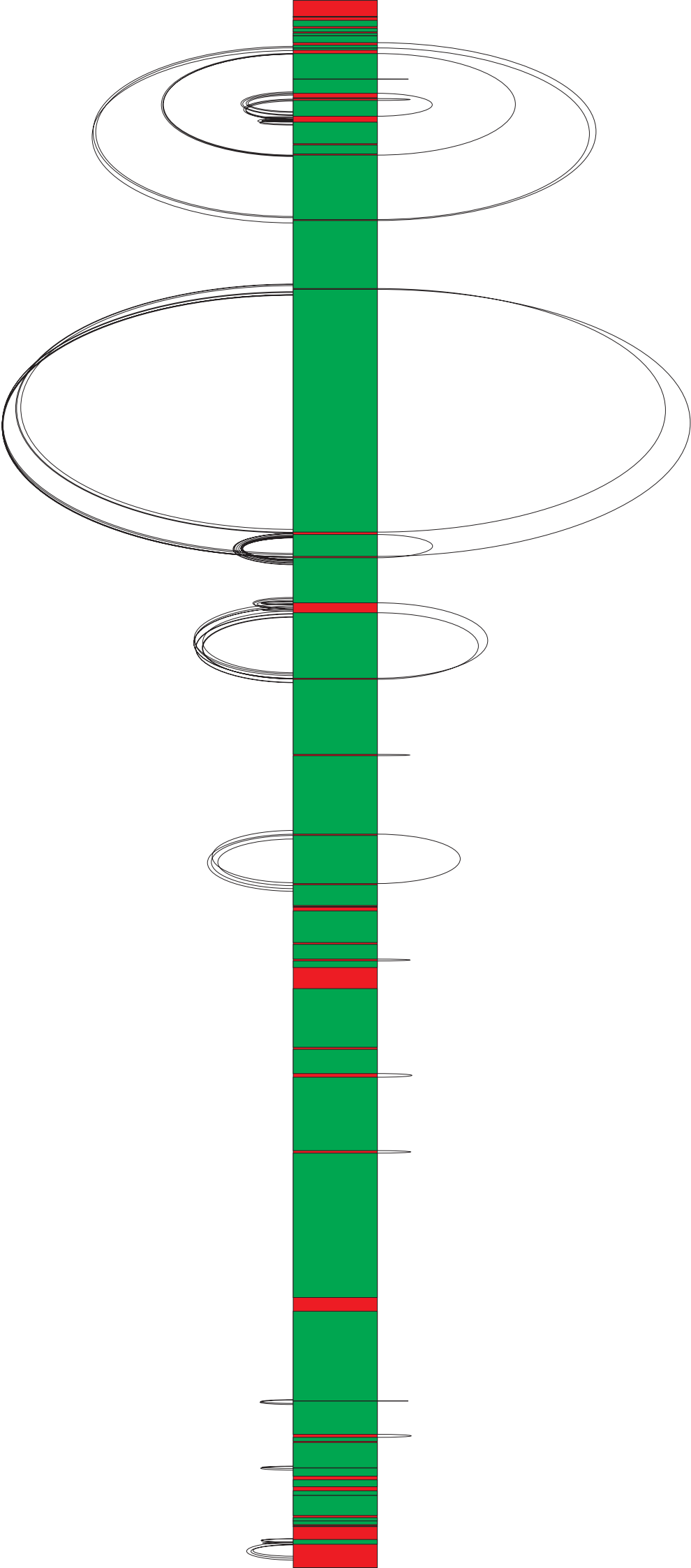

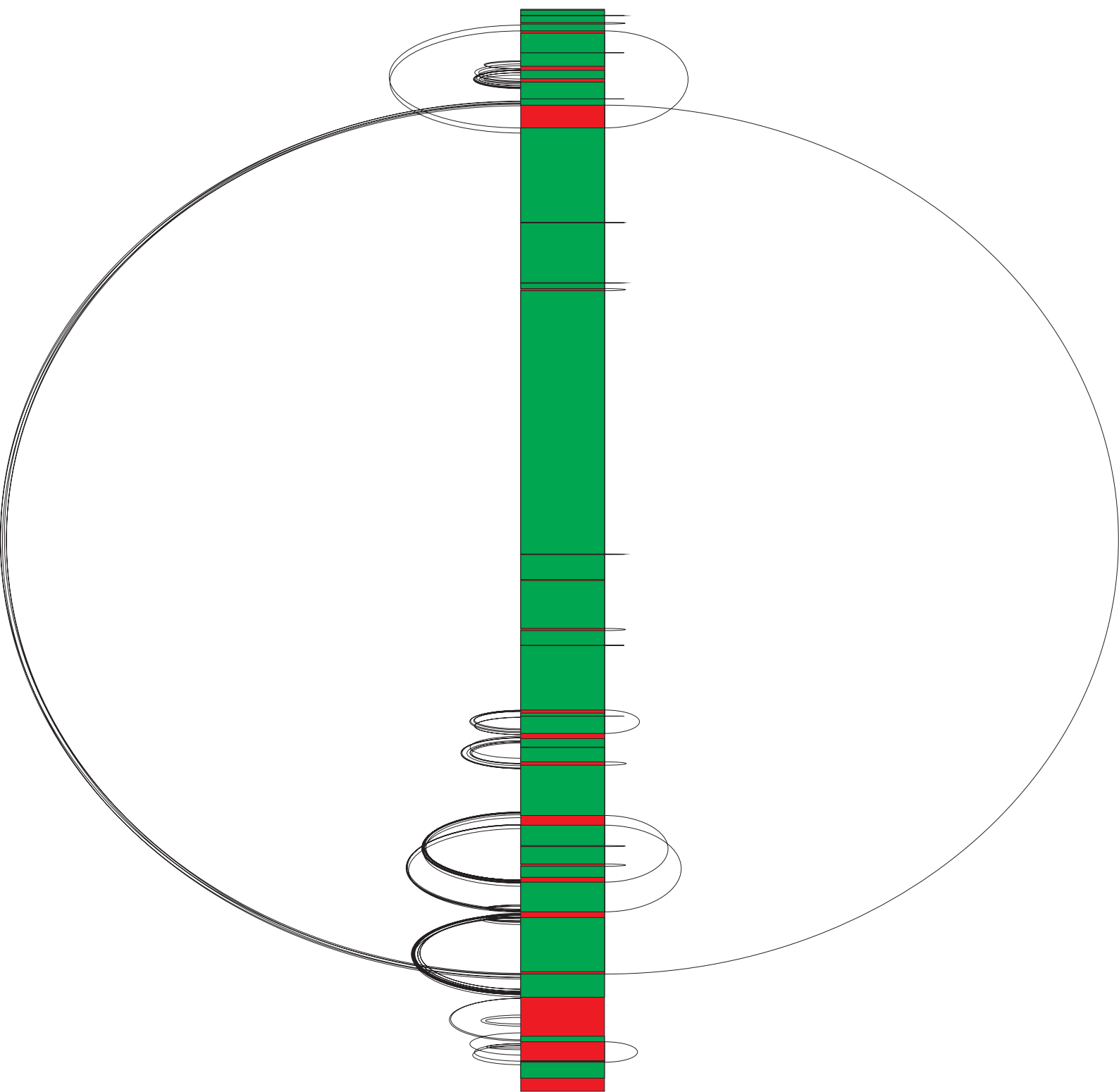

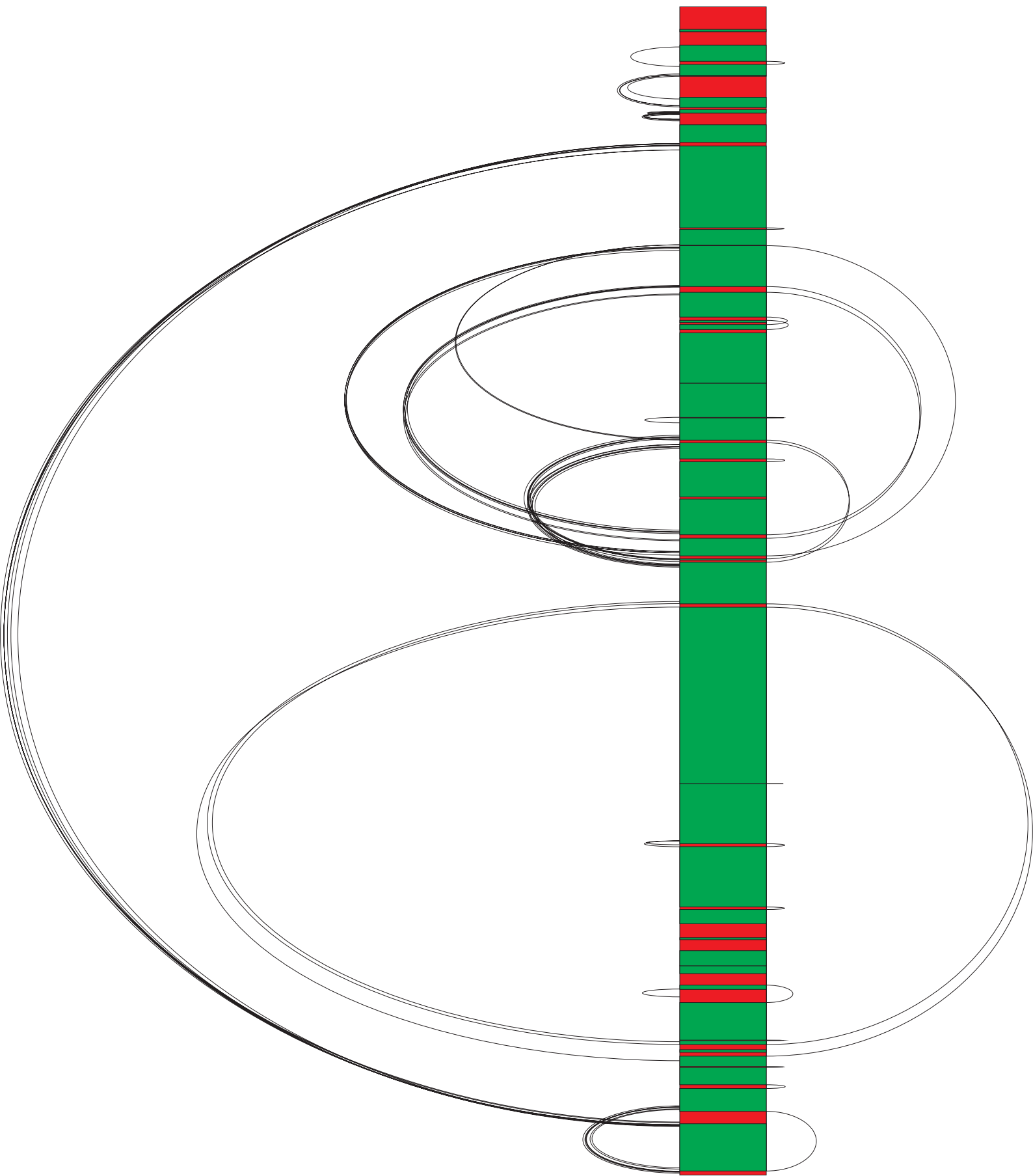

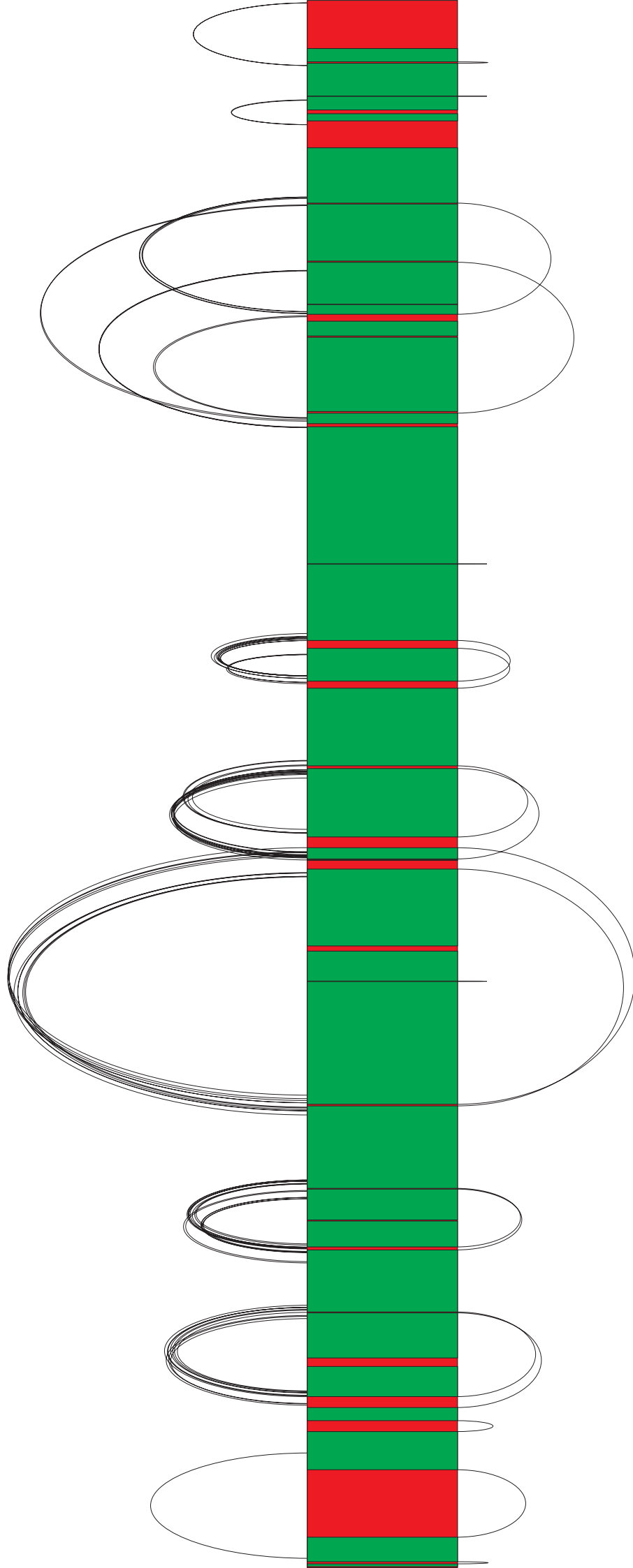

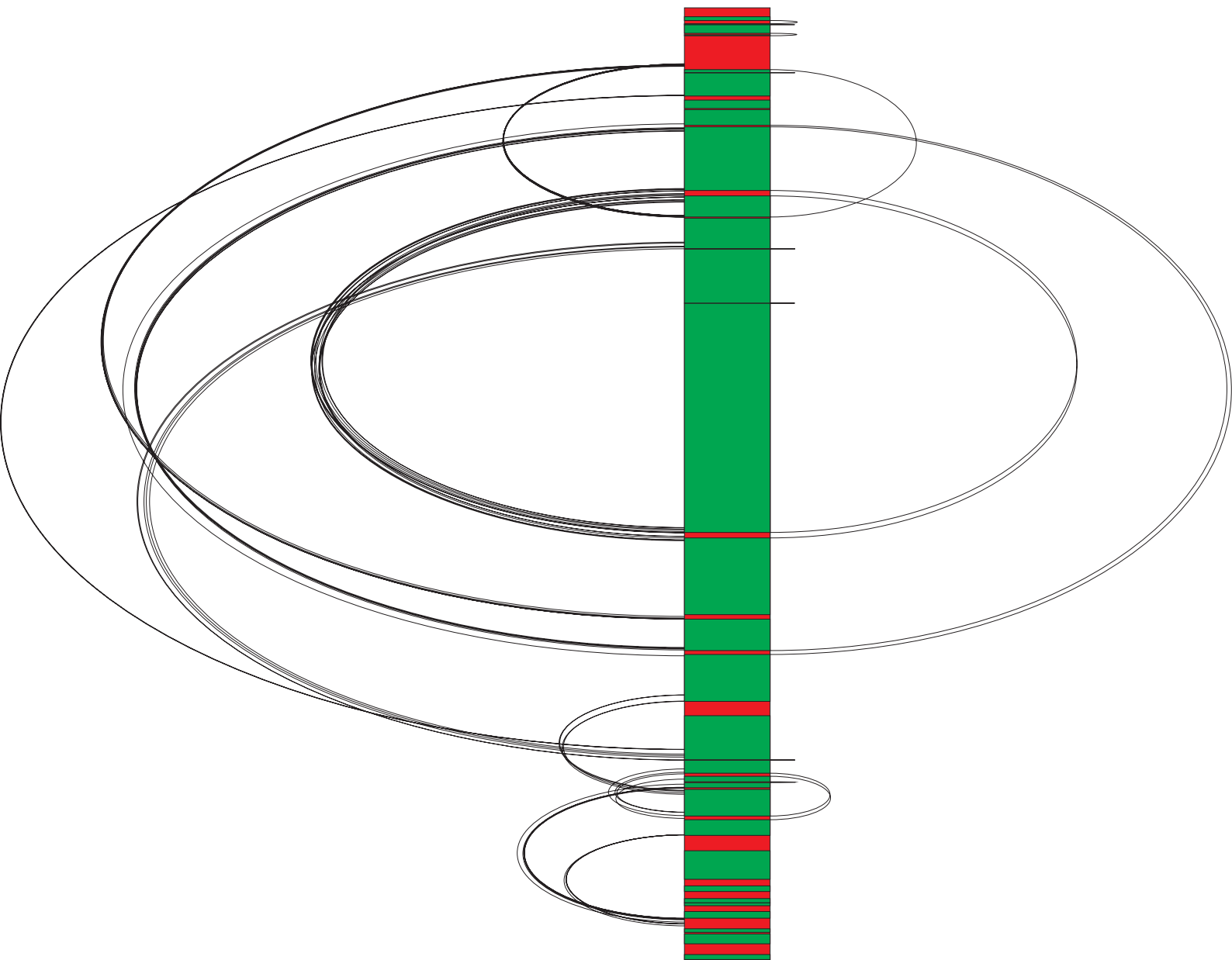

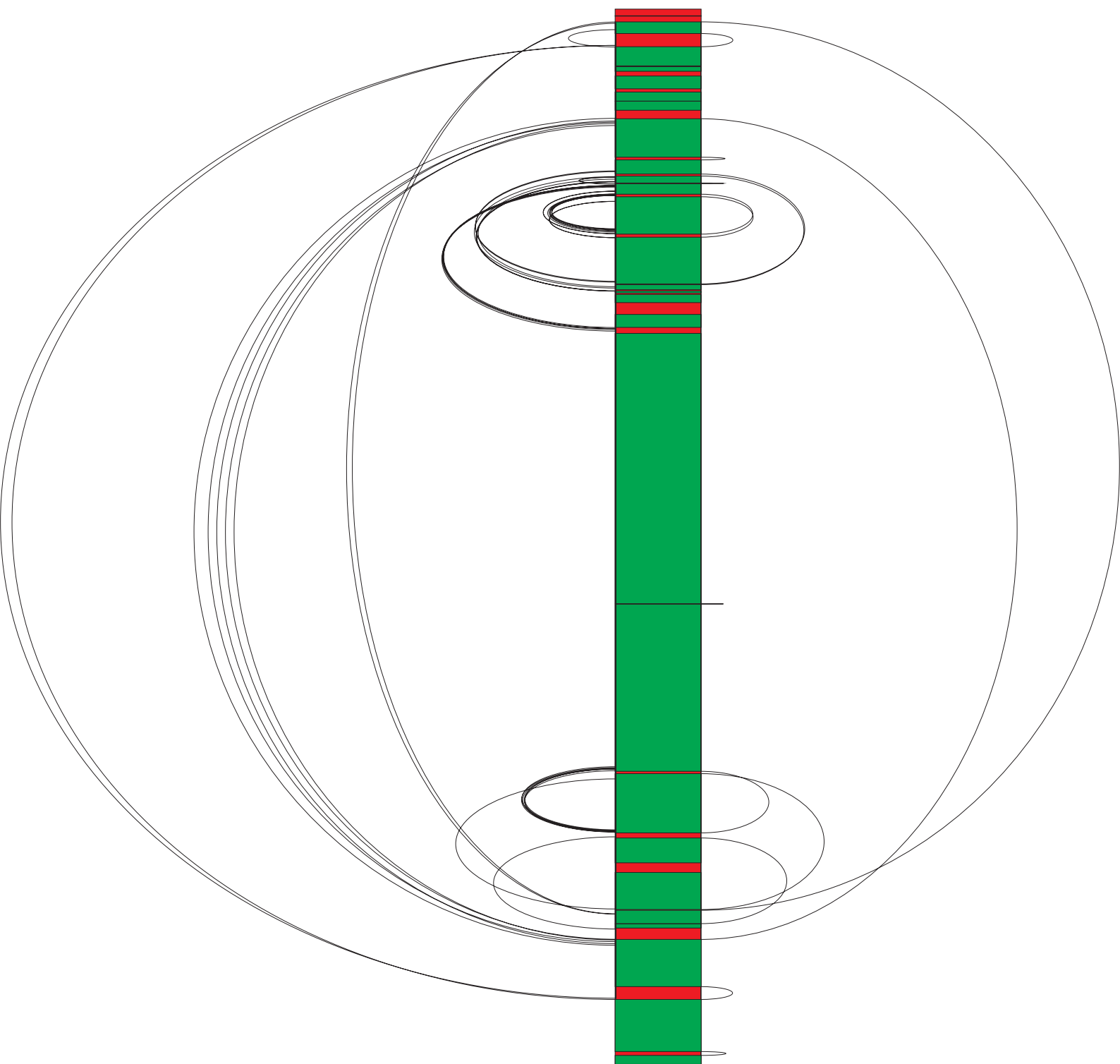

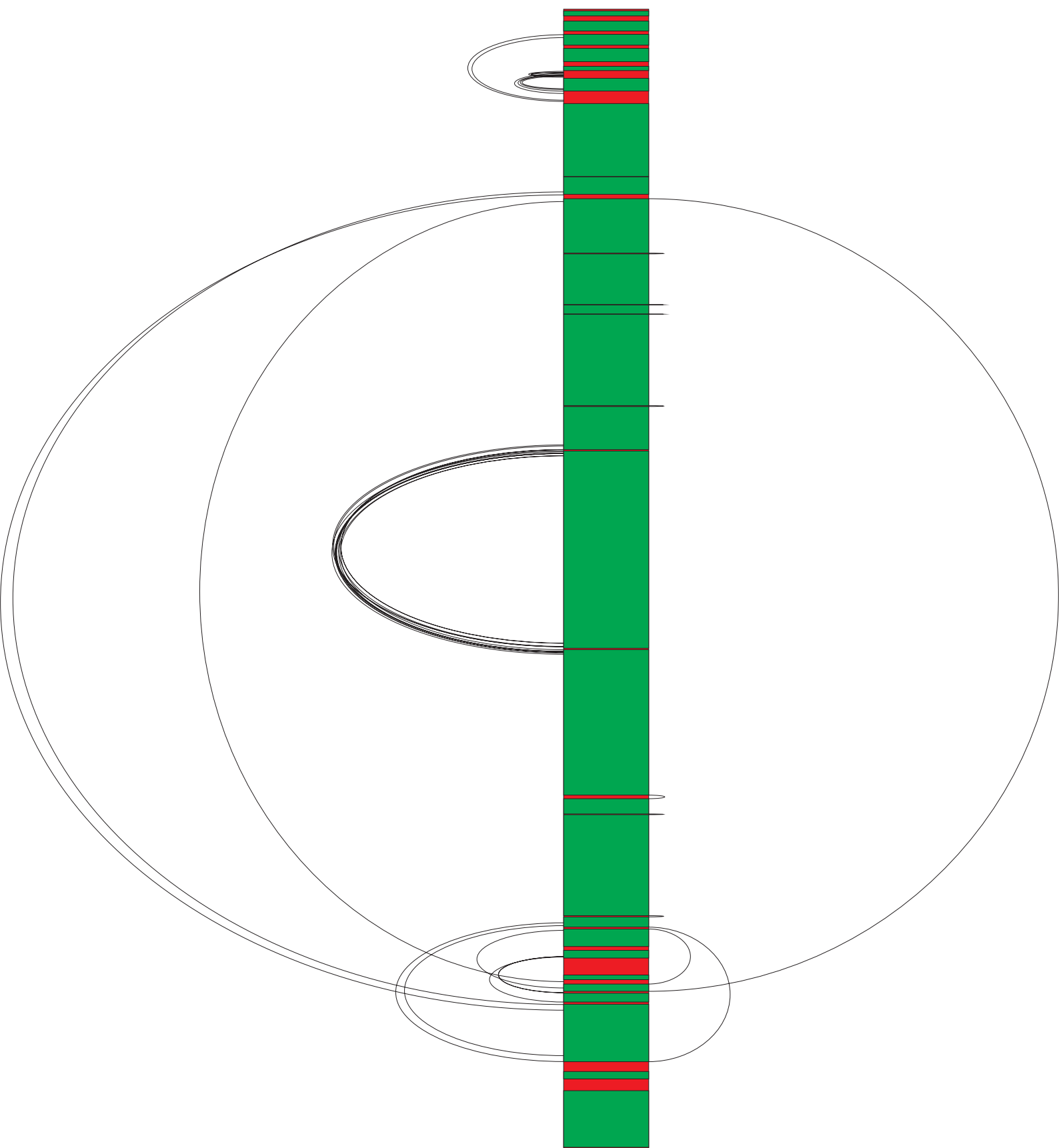

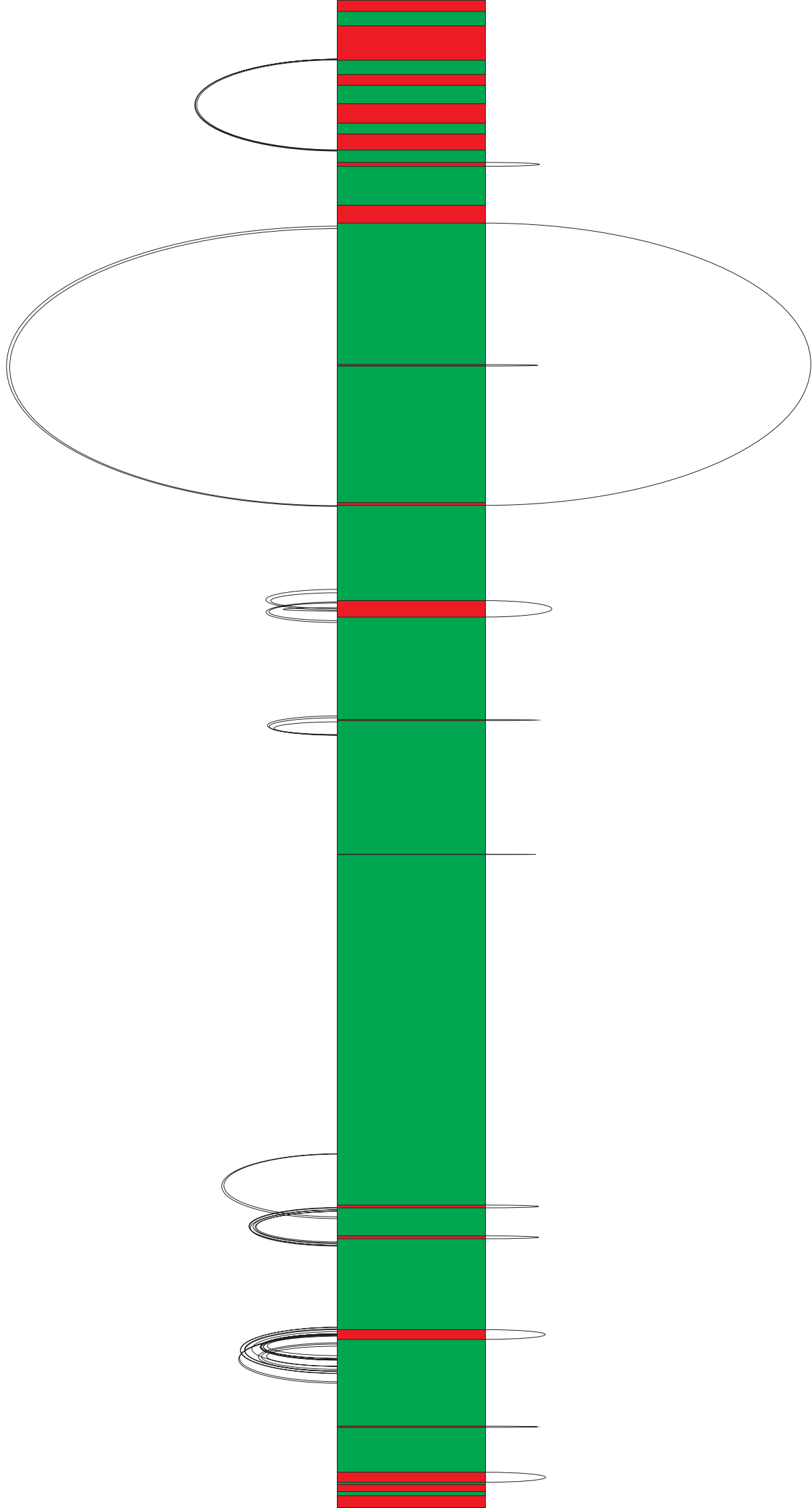

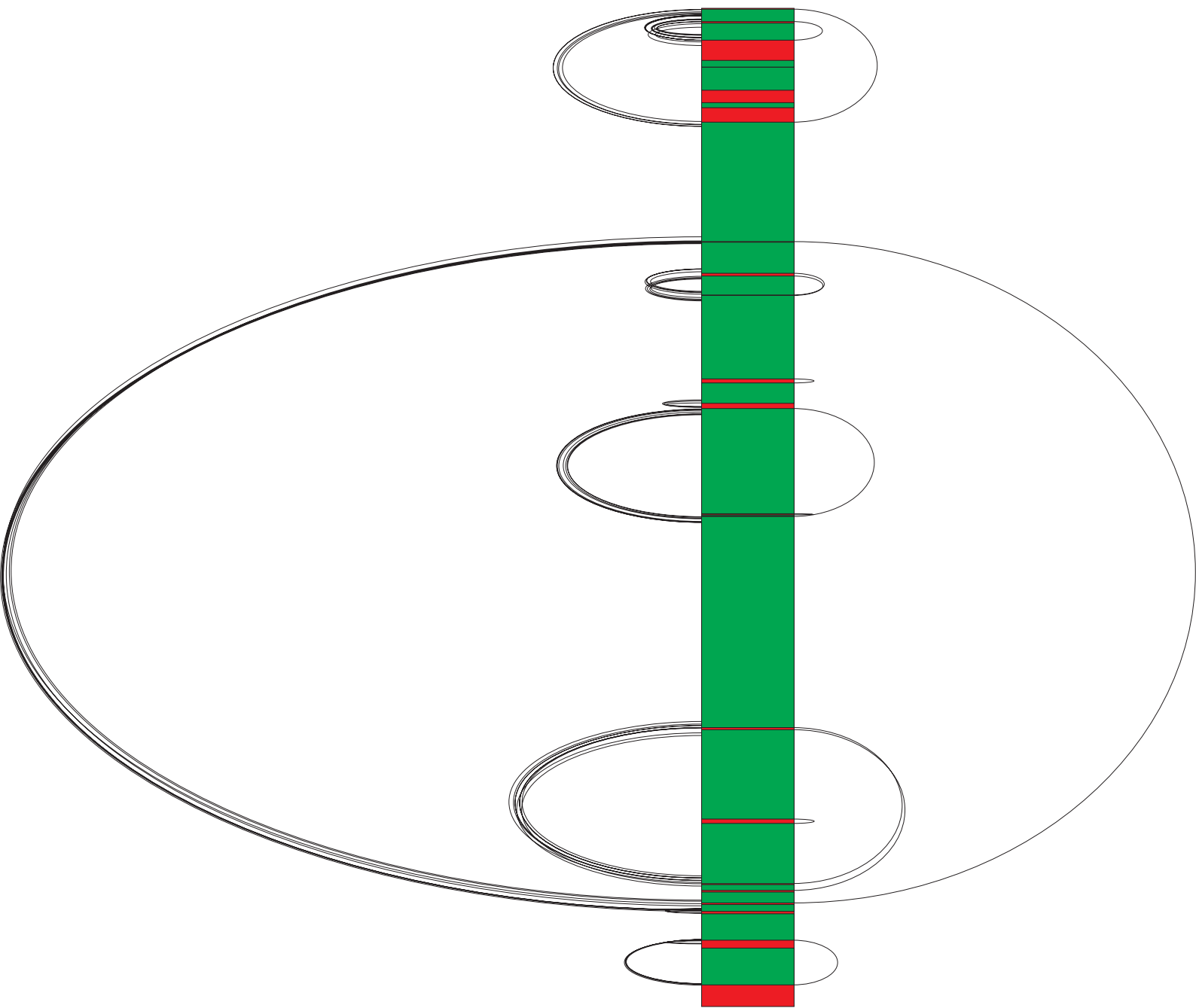

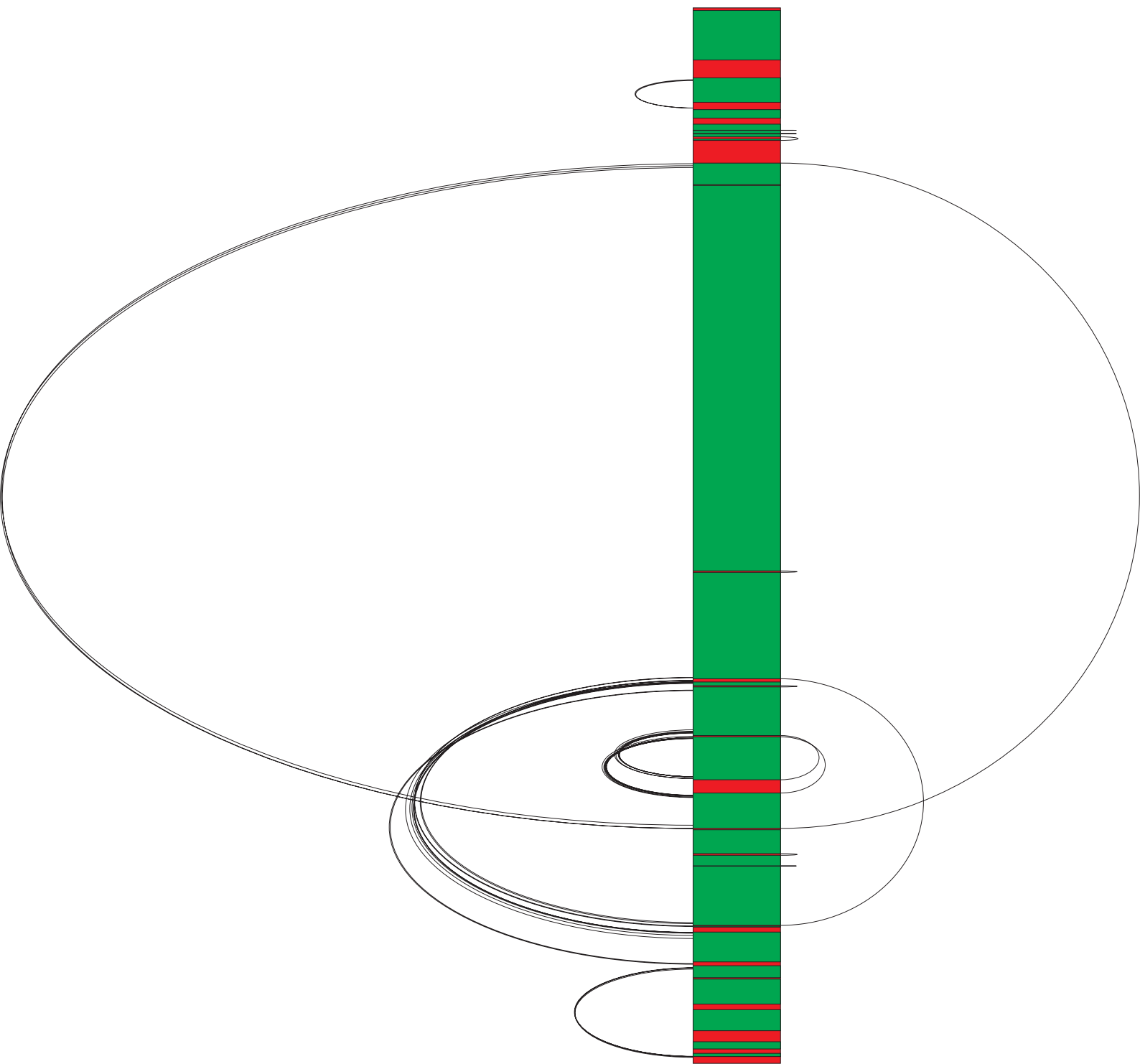

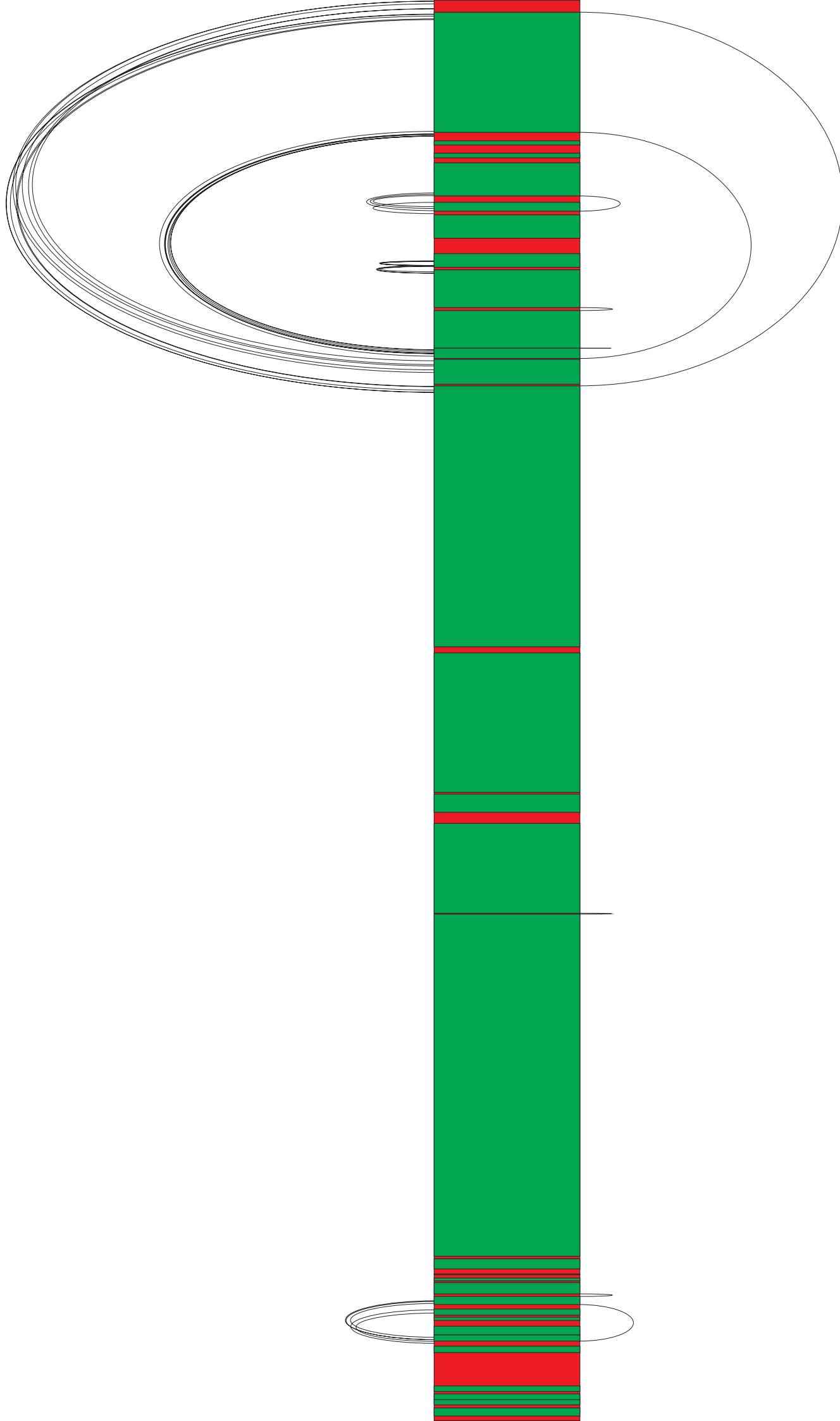

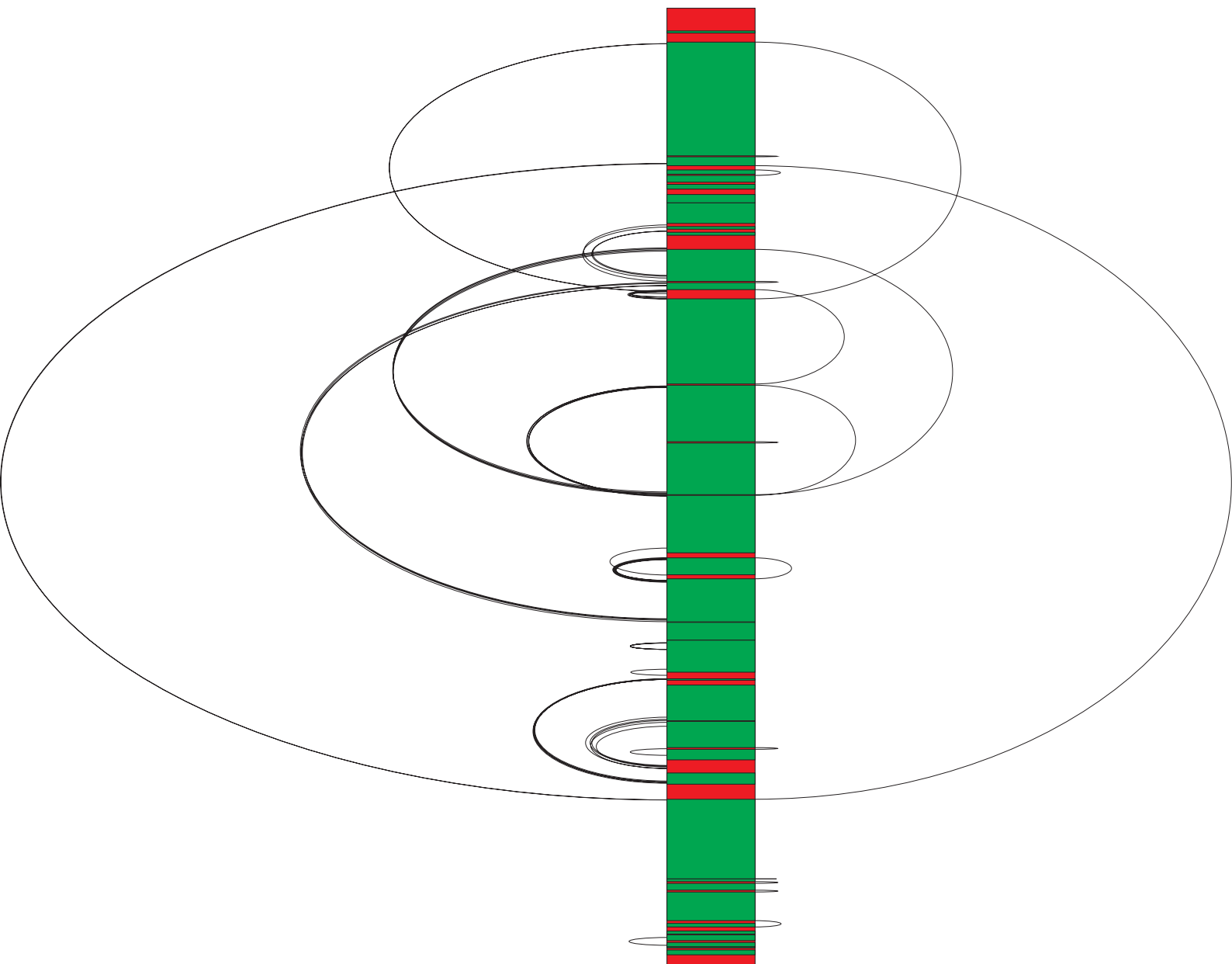

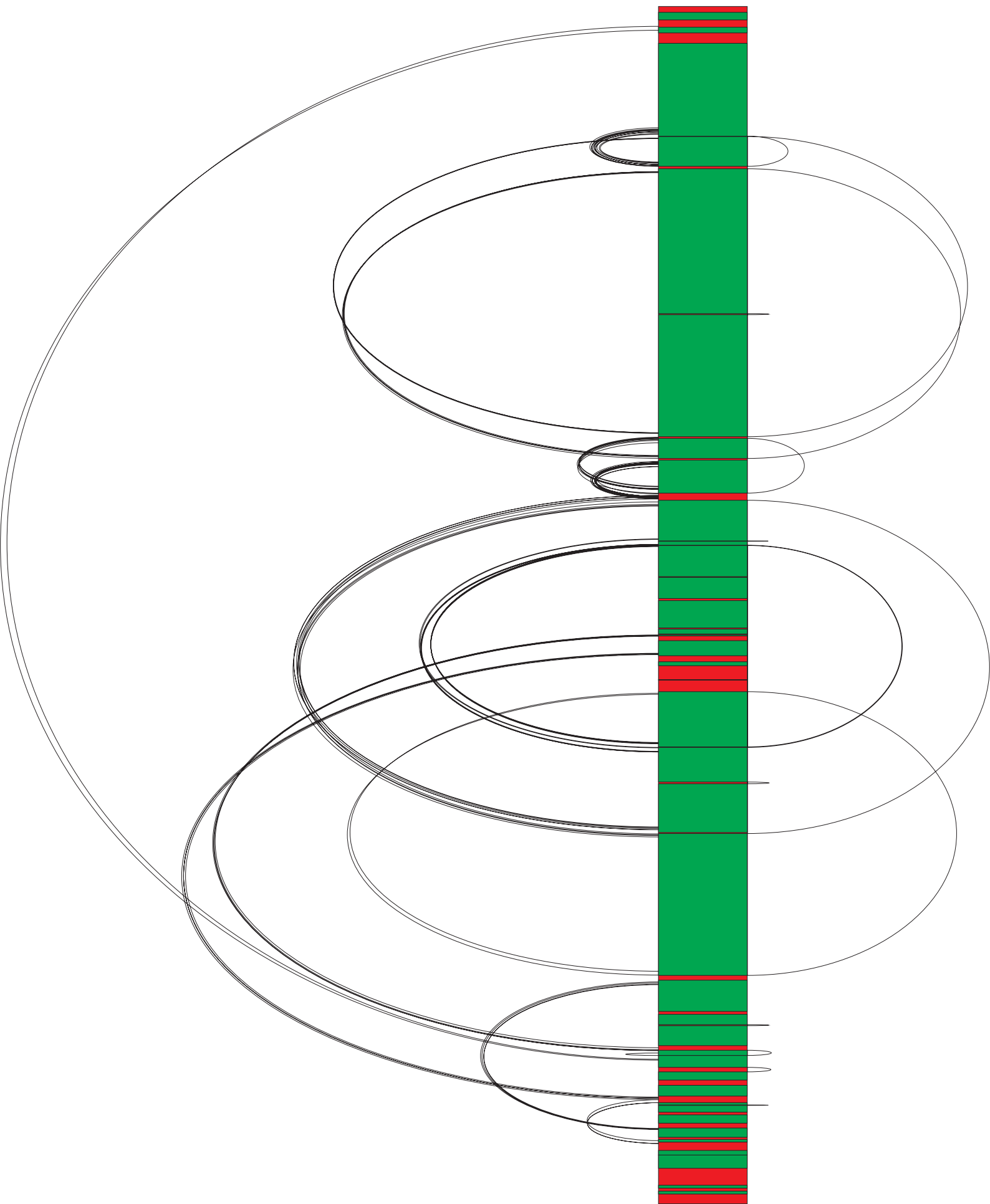

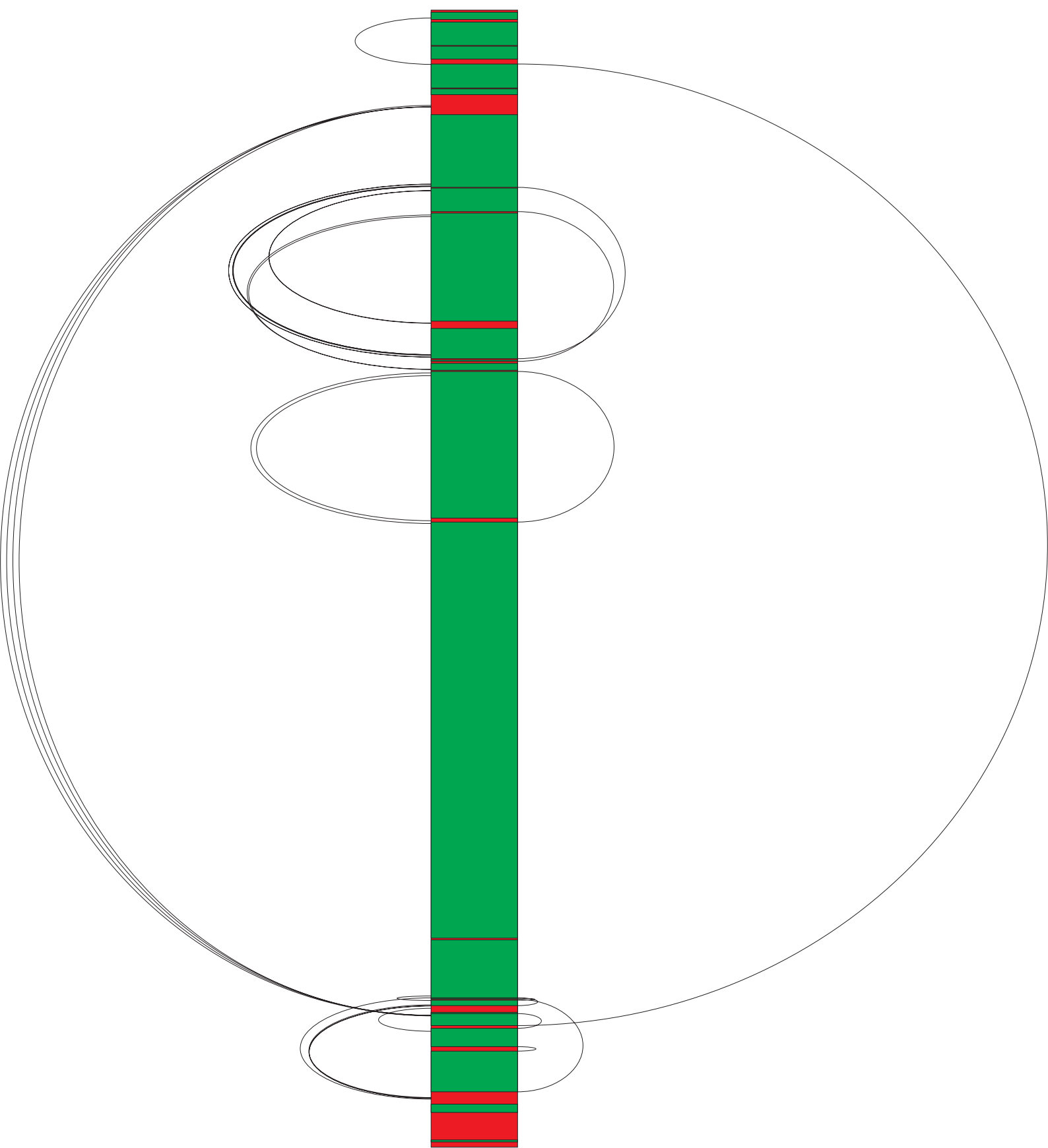

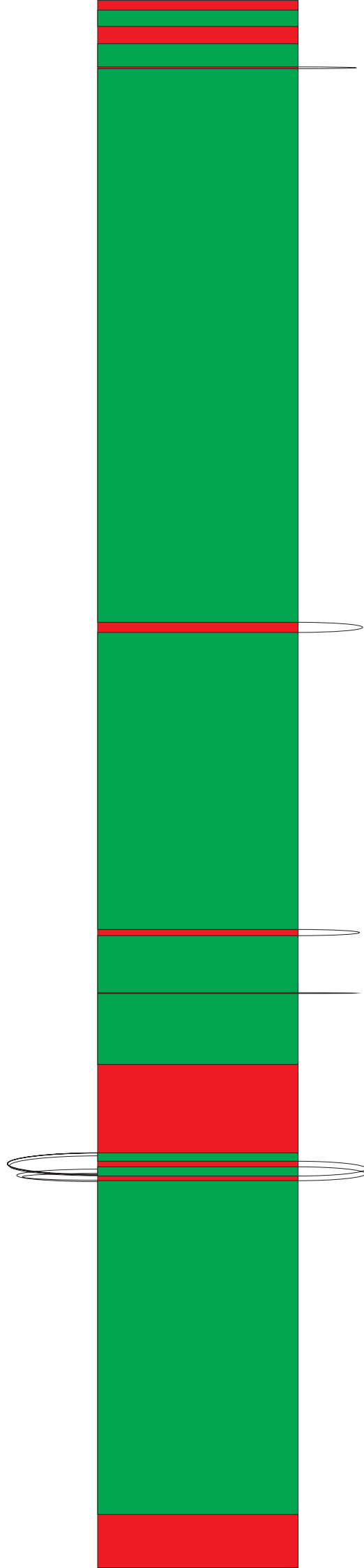

Supplement: Additional file 2 — rearrangements_all_chr. The file contains the 21 stickleback chromosomes (page 1 = chr I; ....; page 21 = chr XXI), showing potential intra-chromosomal rearrangements between sea bass and stickleback (left) and similar rearrangements between medaka and stickleback (right). [file 1471-2164-11-68-S2.PDF]
